# Ly6G^**+**^ inflammatory cells enable the conversion of cancer cells to cancer stem cells in an irradiated glioblastoma model

**DOI:** 10.1038/s41418-019-0282-0

**Published:** 2019-02-25

**Authors:** Hee-Young Jeon, Seok Won Ham, Jun-Kyum Kim, Xiong Jin, Seon Yong Lee, Yong Jae Shin, Chang-Yong Choi, Jason K. Sa, Se Hoon Kim, Taehoon Chun, Xun Jin, Do-Hyun Nam, Hyunggee Kim

**Affiliations:** 1 Department of Biotechnology, College of Life Sciences and Biotechnology, Korea University, Seoul, Republic of Korea; 2 Institute of Animal Molecular Biotechnology, Korea University, Seoul, Republic of Korea; 3 Department of Neurosurgery, Samsung Medical Center, Sungkyunkwan University School of Medicine, Seoul, Republic of Korea; 4 Institute for Refractory Cancer Research, Research Institute for Future Medicine, Samsung Medical Center, Seoul, Republic of Korea; 5 Department of Pathology, College of Medicine, Yonsei University, Seoul, Republic of Korea; 6 Tianjin Medical University Cancer Institute and Hospital, Tianjin, China; 7 Institute of Translational Medicine, The First Affiliated Hospital of Wenzhou Medical University, Wenzhou, China; 8 Department of Health Science & Technology, Samsung Advanced Institute for Health Science & Technology, Sungkyunkwan University, Seoul, Republic of Korea; 9 Department of Medical Engineering, College of Medicine, Korea University, Seoul, Republic of Korea

**Keywords:** Cancer microenvironment, Cancer stem cells

## Abstract

Most glioblastomas frequently recur at sites of radiotherapy, but it is unclear if changes in the tumor microenvironment due to radiotherapy influence glioblastoma recurrence. Here, we demonstrate that radiation-induced senescent glioblastoma cells exhibit a senescence-associated secretory phenotype that functions through NFκB signaling to influence changes in the tumor microenvironment, such as recruitment of Ly6G^+^ inflammatory cells and vessel formation. In particular, Ly6G^+^ cells promote conversion of glioblastoma cells to glioblastoma stem cells (GSCs) through the NOS2-NO-ID4 regulatory axis. Specific inhibition of NFκB signaling in irradiated glioma cells using the IκBα super repressor prevents changes in the tumor microenvironment and dedifferentiation of glioblastoma cells. Treatment with Ly6G-neutralizing antibodies also reduces the number of GSCs and prolongs survival in tumor-bearing mice after radiotherapy. Clinically, a positive correlation exists between Ly6G^+^ cells and the NOS2-NO-ID4 regulatory axis in patients diagnosed with recurrent glioblastoma. Together, our results illustrate important roles for Ly6G^+^ inflammatory cells recruited by radiation-induced SASP in cancer cell dedifferentiation and tumor recurrence.

## Introduction

It has been reported that glioblastoma (GBM) inevitably recurs at the initial tumor site where surgery and radiotherapy were performed [1–3]. After irradiation of solid tumors, including GBM, tumor-associated macrophages (TAMs) and monocytes are recruited to the tumor and promote vessel formation, tumor progression, and recurrence [4, 5]. In tumors, Ly6G-expressing (Ly6G^+^) inflammatory cells, including granulocytic myeloid-derived suppressor cells (G-MDSCs) and tumor-associated neutrophils (TANs), increase secretion of various cytokines and chemokines that promote tumor growth, metastasis, angiogenesis, and suppression of anti-tumor immunity [6, 7]. The relationship between the changes in the radiation-induced tumor microenvironment and tumor-associated myeloid cells, however, remains poorly understood.

Many studies have demonstrated that glioblastoma stem cells (GSCs) occur as small populations within tumors and possess tumor-initiating ability and properties such as self-renewal and multi-lineage differentiation, similar to normal stem cells [8, 9]. As GSCs are resistant to chemotherapy and radiotherapy, their presence is considered to be a determinant of tumor recurrence [10]. GSCs are located in restricted microenvironments, such as perivascular and hypoxic/necrotic niches, allowing these cells to maintain self-renewal ability [11, 12]. The tumor microenvironment plays an important role not only in maintaining GSC features, but also in the dedifferentiation of non-stem glioma cells into GSCs [13].

Senescent cells exist in a state of irreversible cell cycle arrest; however, these cells are metabolically active and exhibit factors related to the senescence-associated secretory phenotype (SASP). The SASP includes soluble factors such as growth factors, cytokines, interleukins (ILs), secreted proteases, and other factors that can promote tumor progression and modify the tumor microenvironment [14]. For example, the SASP includes IL6 and IL8, which increase and maintain GSC self-renewal [15, 16], and SDF-1, which recruits TAMs and increases vasculogenesis [17]. Our recent study demonstrated that irradiation-induced senescence in glioblastoma cells increased the expression of SASP and promoted tumor progression in vivo [18]. There is, however, little evidence of a molecular mechanism linking irradiation-induced SASP with tumor recurrence, particularly in the context of SASP and GSCs.

Here, using a stem cell fate-tracking system (OCT4-promoter-reporter), we demonstrated that the radiation-induced SASP leads to changes in the tumor microenvironment, including Ly6G^+^ inflammatory cell recruitment and vessel formation, which result in the conversion of non-stem glioblastoma cells to GSCs. Additionally, we revealed that interventions targeted toward this tumor microenvironment prevented tumor recurrence and reduced GSC dedifferentiation.

## Materials and methods

### Cells and culture conditions

The human glioma cell lines U87MG (*p53*wt, *PTEN*mut, *p14ARF/p16*del) and LN229 (*p53*mut, *PTEN*wt, *p14ARF/p16*del) were purchased from the American Type Culture Collection (ATCC, Manassas, VA, USA). U87MG, LN229, and BV2 cells (murine microglia) were cultured in high-glucose Dulbecco’s Modified Eagle medium (DMEM, Lonza, Basel, Switzerland) supplemented with 10% fetal bovine serum (FBS, Serana, Bunbury, Australia), 1% penicillin/streptomycin (Lonza, Basel, Switzerland), and 2 mM l-glutamine (Lonza) at 37 °C with 5% CO_2_ and 95% humidity. For stem cell culture, cells were incubated with DMEM/F12 (Lonza) supplemented with B27 (Invitrogen, Carlsbad, CA, USA), 20 ng/mL EGF (R&D Systems), and 20 ng/mL bFGF (R&D Systems). EGF and bFGF were replaced every 3 days.

Human retinal endothelial cells (HRECs) were grown in endothelial cell growth medium (EGM-2, Lonza). The human leukemic cell line HL-60 was cultured in Roswell Park Memorial Institute medium (RPMI-1640, Lonza) supplemented with 5% FBS, 1% penicillin/streptomycin, and 2 mM l-glutamine. For differentiation into neutrophil-like cells, HL-60 cells were incubated in RPMI culture medium containing 1.25% DMSO for 5 days [19].

### Plasmid construction and gene transfection

The hOCT4-p-eGFP vector was generously provided by Dr. Wei Cui (Imperial College London, London, UK) [20]. The pLL-CMV-IκBα mutant-puro vector was cloned with the insert from pBabe-GFP-IκBα-mutant (super repressor) that was a gift from Dr. William Hahn (Addgene plasmid # 15264) [21]. IκBα-mutant cDNA was cloned into the pLL-CMV-puro lentiviral vector.

U87MG and LN229 cells were transfected with the phOCT4-p-eGFP vectors using a microporator and the Neon Transfection System (MKP10096, Invitrogen) according to manufacturer instructions. GFP-N cells were sorted using a fluorescence-activated cell sorting (FACS) system (FACS Aria, BD bioscience) a total of 3 times to eliminate contamination. U87MG and LN229 cells were infected with lentivirus produced from the 293FT cell line (Life Technologies) that was transfected with a lentiviral vector (pLL-CMV-puro, pLL-CMV-IκBα mutant-puro, pLL-CMV-ID4-puro and pCDH-CMV-EF1-DsRed) and packaging vectors (3rd generation: pMDLg/pRRE, pRSV-Rev, and pMD2.G).

### ^137^Cs γ-ray irradiation

^137^Cs γ-ray irradiation at a dose rate of 2.04 Gy/min for a total dose of 20 Gy was conducted using an IBL 437C (CIS Bio-International, Codolet, France).

### Subcutaneous and orthotopic glioma cell implantation

For subcutaneous models, 1 × 10^6^ or 1 × 10^5^ U87MG/LN229-OCT4-p-GFP-negative cells (GFP-N) or 5 × 10^5^ irradiated U87MG/LN229-DsRed cells (I-Red) were injected into nude mice (BALB/c nu/nu). Also, 1 × 10^5^ U87MG/LN229-GFP-N cells were co-injected with 5 × 10^5^ U87MG/LN229-I-Red cells. Mice were sacrificed when the tumor size exceeded 1500 mm^3^, and the tumors were harvested. To determine the time-frame for inflammatory cell recruitment, 1 × 10^6^ U87MG and LN229 cells were injected alone or co-injected with 2 × 10^6^ I-Red cells mixed with Matrigel (354234, BD bioscience).

For orthotopic implantation, 5 μL of 1 × 10^5^ U87MG-GFP-N and LN229-GFP-N cells were stereotactically injected into the nude mice brain (coordinates: 2 mm right and 1 mm rostral of the bregma and 3 mm depth from the surface of the skull [22]). Five microliters of 1 × 10^5^ U87MG-GFP-N and LN229-GFP-N cells were co-injected with 2 × 10^5^ irradiated U87MG-puro-DsRed or IκBα-DsRed and LN229-puro-DsRed or IκBα-DsRed cells, respectively. All mouse experiments were approved by the Animal Care Committee of Korea University in accordance with government and institutional guidelines and Korean regulations.

### Tumor dissociation for single-cell analysis

The tumors were minced into 2–4 mm fragments and then incubated with trypsin for 30 min at 37 °C. The fragments were filtered through a 40-μm nylon mesh cell strainer (CD1-1KT, Sigma-Aldrich, St. Louis, MO, USA). The released cells were centrifuged at 1200 rpm for 3 min and incubated in culture media at 37 °C with 5% CO_2_ and 95% humidity [23]. These single cells were incubated with Ly6G antibody (551459, BD Bioscience, San Jose, CA, USA), followed by anti-rat biotin (BA-9400, VECTOR laboratories, CA, USA), and streptavidin-PE (554061, BD Pharmingen, San Diego, CA, USA). Tumor-derived Ly6G^+^ cells, GFP-positive cells, and GFP-negative cells were sorted using a fluorescence-activated cell sorting (FACS) system (FACS Aria, BD bioscience).

### Treatment with anti-Ly6G antibody following fractionated irradiation of orthotopic xenograft mouse models

For mouse irradiation, 3 × 10^5^ U87MG and LN229 glioma cells were injected into the nude mice brain. At 3 weeks post injection, the anesthetized mice were placed in a lead shielding device, and then the whole brains of mice were irradiated 5 times at 2 Gy per day (2 Gy × 5). Rat anti-Ly6G antibodies (clone 1A8, BE0075-1, BioXcell, West Lebanon, NH, USA) or rat IgG isotype controls (clone 2A3, BE0089, BioXcell) dissolved in 100 μL PBS were used for Ly6G^+^ inflammatory cell depletion. Antibodies were administered at 100 μg every 72 h via intraperitoneal injection after exposure to radiation [6]. For the experimental group in which the antibody was administered before exposure to radiation, 300 μg of antibody was used prior to irradiation, and then 100 μg was used as described above. Blood samples were taken during antibody injections for FACS analysis.

### Hematoxylin–eosin staining and immunofluorescence in tumor tissues

Tumor-bearing mice were perfused with PBS and 4% paraformaldehyde (PFA, 158127, Sigma-Aldrich). Obtained tumor tissues were embedded in paraffin, sectioned (4μm in thickness), and placed on glass slides. After deparaffinization and hydration, the tissue slides were treated with hematoxylin (Merck, Darmstadt, Germany) for 5 min and rinsed with tap water. Next, tissue slides were dipped 10–15 times in acidic alcohol and rinsed again in tap water. All slides were incubated in an eosin solution (109844, Merck) for 30 s, followed by washing with distilled water.

After deparaffinization and hydration, the tissue slides were stained with primary antibodies against Nestin (1:500; MAB1259, R&D Systems, Minneapolis, MN, USA), CD133 (1:50; 130-092-395, Miltenyi Biotec, Auburn, CA, USA), OCT4 (1:200; sc-8629, Santa Cruz Biotechnology, Dallas, TX, USA), Ly6G (1:500, BD Bioscience), IBA1 (1:500; 019-19741, WAKO, Richmond, VA, USA), F4/80 (1:200, MF48000, Invitrogen), CD31 (1:500; sc-1506, Santa Cruz Biotechnology), GFP (1:1000; ab290, Abcam, Cambridge, UK), RFP (1:500; 600-401-379, Rockland Immunochemicals, Gilbertsville, Pennsylvania, USA), ID4 (1:500; ab20988, Abcam), NOS2 (1:500; 610328, BD Bioscience), and c-CASP3 (1:500; 9661, Cell Signaling Technology, Danvers, MA, USA) for 12 h at 4 °C. Cells were washed twice with PBS and incubated with fluorescence-conjugated secondary antibody (Invitrogen) for 1 h at 18–22 °C. Nuclei were then stained with DAPI (1 μg/mL) for 5 min. Triplicate images for each mouse were obtained using an Epifluorescence and Brightfield microscope (Axioimager M1, Carl Zeiss, MA, USA) and quantified with MetaMorph software (Molecular Devices, CA, USA). To analyze the GSC population affected by neutrophils, we quantified the number of the stem cell marker-positive cells located within 100 μm diameter regions of the Ly6G-positive cells.

### Sphere-forming assay

For sphere-forming assays, cells were plated at a density of 200 cells/24-well plate. For in vitro limiting dilution assays, cells were plated in 96-well plates with decreasing numbers of cells per well (200, 100, 50, 20, and 10). The tumor sphere numbers were determined after 14 days. Extreme limiting dilution analysis was performed using software available at http://bioinf.wehi.edu.au/software/elda/.

### FACS analysis

Cells were harvested and washed twice with PBS. Cells were fixed with 4% PFA for 15 min, then were incubated with 3% bovine serum albumin (BSA, 82-100-6, Millipore, Burlington, MA, USA) in PBS to detect membrane proteins, or they were incubated with 3% BSA in PBS containing 0.1% saponin (S7900, Sigma-Aldrich) to detect intracellular proteins. Antibodies used for stem cell markers analysis included Nestin (1:200; R&D Systems), CD133 (1:10; Miltenyi Biotec), OCT4 (1:200; Santa Cruz Biotechnology), SOX2 (1:200; AF2018, R&D systems), and NANOG (1:200; ab21624, Abcam). The biotinylated secondary antibodies (VECTOR laboratories) were incubated for 30 min, and then streptavidin PE (BD Bioscience) was added for 10 min. Antibodies used for inflammatory cells analysis were CD45.2-PerCP-Cy5.5 (1:200; 552950, BD Bioscience), CD45-APC (1:200; 559864, BD Bioscience), CD11b-FITC (1:200; 11-0112-82, eBioscience, San Diego, CA, USA), F4/80-PE (1:200; MF48004, Invitrogen) and Ly6G-PE (1:200, 551461, BD Bioscience).

For GFP induction analysis, GFP-N cells (5 × 10^4^ cells) were co-cultured with N-Red or I-Red cells (10^5^ cells) under stem cell culture conditions on days 1−4. The fluorescence intensities were measured by flow cytometry (FACS Verse, BD Bioscience).

### Detection of radioresistance

For colony-formation assays, 700 cells were irradiated at 0, 2, or 3 Gy and seeded in triplicate wells of 6-well plates. After 2 weeks, the colonies were fixed with 4% PFA, stained with crystal violet, and counted. For the MTS assay, 3000 cells were irradiated at 0, 2, 4, or 6 Gy and seeded in triplicate wells of 96-well plates. After 3 days, EZ-Cytox solution (DOGEN, Seoul, Korea) was added to each well, and then cells were incubated for 4 h. The light absorbance was determined at 450 nm by the PowerWaveXS microplate-reader (Bio-Tek Instruments Inc., VT, USA).

### Tubule formation assay

For CM collection, irradiated glioma cells (0 and 20 Gy) were cultured in Endothelial Cell Basal Medium (EBM, Lonza) for 1 day, then harvested and filtered through 0.2 μm filters (16534, Sartorius Stedim Biotech, Goettingen, Germany). We performed tubule formation assays using the in vitro angiogenesis assay kit (ECM625, Chemicon, Temecula, CA, USA) according to manufacturer instructions. Briefly, HRECs (7 × 10^3^ cells/well) were seeded on Matrigel in EGM-2, EBM, or CM from irradiated or non-irradiated cells (0 and 20 Gy) at 37 °C for 5 h. Three random view fields per well were examined, and the tubes were counted.

### Invasion and migration assays

For CM collection, irradiated glioma cells (0 or 20 Gy) were cultured in DMEM or RPMI-1640 containing 1% FBS, followed by harvesting and filtering through 0.2μm filters. For transwell invasion assays of BV2, the transwell inserts (3422, Corning costar, Cambridge, MA, USA) were coated with Matrigel for 4 h in an incubator at 37 °C. Then, BV2 cells (3 × 10^4^ cells/well) suspended into serum-free DMEM were seeded into the transwell inserts. The CM from irradiated and non-irradiated cells cultured in DMEM with 1% FBS was plated in the lower chamber. After 24 h, invasive cells were counted. For transwell migration assays of dHL-60 cells, dHL-60 cells (3 × 10^4^ cells/well) suspended in serum-free RPMI-1640 were seeded into the transwell inserts (3421, Corning Costar). The CM from irradiated or non-irradiated cells cultured in RPMI-1640 with 1% FBS was placed in the lower chamber. After 4 h, the migrated cells were counted.

### Nitrite level determination by 4,5-diaminofluorescein

Conditioned supernatants of dHL-60 cells pre-incubated with CM from irradiated glioma cells (0 or 20 Gy) were collected and filtered through 0.2 μm filters (16534, Sartorius Stedim Biotech). Fifty microliters of conditioned supernatant or nitrite standards were added in triplicate for each condition into a 96-well plate. Twenty-five microliters of 30 µM 4,5-diaminofluorescein (DAF-2; Cayman Chemical, Ann Arbor, MI, USA) in PBS was added and incubated for 15 min at room temperature in the dark. The samples were acidified with 50 µL of 1 M HCl and incubated for 15 min, then neutralized with 50 µL 1.5 M NaOH. Fluorescence was measured at excitation and emission wavelengths of 488 and 525 nm, respectively, using a microplate reader (Hidex, Turku, Finland). A standard curve was created using the fluorescence from the nitrite standards (Cayman Chemical) as described previously [24].

### SA-β-gal staining

To detect cell senescence, non-irradiated or irradiated GBM cells and xenograft tumor sections (4 μm thick) were stained using a Senescence β-Galactosidase Staining Kit (9860, Cell Signaling Technology) according to manufacturer instructions. Briefly, after washing the plate or slides with PBS, a 1× fixative solution was added to each well or slide for 15 min at room temperature. Next, the plates or slides were rinsed with PBS, and then β-galactosidase staining solution was added. The plates or slides were incubated overnight at 37 °C in a dry incubator (no CO_2_). After incubation, the slides were stained with Nuclear fast red (NFR; N3020, Sigma-Aldrich) for 5 min and rinsed with tap water. Cells and tissues were analyzed using an inverted fluorescence microscope (Axio Observer D1, Carl Zeiss) to detect the blue staining.

### Quantitative reverse transcription-polymerase chain reaction (qRT-PCR)

qRT-PCR was performed to determine mRNA levels. Total RNA was isolated from cells using TRIzol Reagent (Invitrogen) according to manufacturer instructions. RNA (1 μg) that had been treated with RNase-free DNase was utilized as a template for synthesizing complementary DNA (cDNA) using the RevertAid First Strand cDNA Synthesis Kit (Thermo Scientific, Waltham, MA, USA) according to manufacturer instructions. qRT-PCR analysis was performed using Takara Bio SYBR Premix Ex Taq and CFX096 (Bio-Rad, Hercules, CA, USA). The expression level of each target gene was normalized to that of *18S* rRNA. The primer sequences were human *18S* rRNA forward: 5′-CAGCCACCCGAGATTGAGCA-3′, reverse: 5′-TAGTAGCGACGGGCGGTGTG-3′; human *MCP1* forward: 5′-CCCAAACTCCGAAGACTTGA-3′, reverse: 5′-CAAAACATCCCAGGGGTAGA-3′; human *GRO1* forward: 5′-AATCCAACTGACCAGAAGGG-3′, reverse: 5′-CATTAGGCACAATCCAGGTG-3′; human *IL6* forward: 5′-CCTGAACCTTCCAAAGATGGC-3′, reverse: 5′-TTCACCAGGCAAGTCTCCTCA-3′; human *IL8* forward: 5′-GCTCTGTGTGAAGGTGCAGT-3′, reverse: 5′-ACTTCTCCACAACCCTCTGC-3′; human *IL1α* forward: 5′-CAGCCAGAGAGGGAGTCATT-3′, reverse: 5′-GGAGTGGGCCATAGCTTACA-3′; human *IL1β* forward: 5′-CCCAACTGGTACATCAGCAC-3′, and reverse: 5′-GGAAGACACAAATTGCATGG-3′; human *SOX2* forward: 5′-CAAGATGCACAACTCGGAGA-3′, and reverse: 5′- CGGGGCCCGTATTTATAATC-3′; human *OCT4* forward: 5′-GACAACAATGAGAACCTTCAG-3′, and reverse: 5′-TTCTGGCGCCGGTTACAGAAC-3′; human *NANOG* forward: 5′-ATAGCAATGGTGTGACGCAG-3′, and reverse: 5′-GATTGTTCCAGGATTGGGTG-3′; human *Nestin* forward: 5′-AACAGCGACGGAGGTCTCTA-3′, and reverse: 5′-TTCTCTTGTCCCGCAGACTT-3′; human *CD133* forward: 5′-TTCACCTGCAGAACAGCTTC-3′, and reverse: 5′-CTGTCTATTCCACAAGCAGCA-3′; mouse *Ccl2* forward: 5′-GCATCTGCCCTAAGGTCTTC-3′, and reverse: 5′-AAGTGCTTGAGGTGGTTGTG-3′; mouse *Ccl3* forward: 5′-TCTCCTACAGCCGGAAGATT-3′, and reverse: 5′-GCCGGTTTCTCTTAGTCAGG-3′; mouse *Tnfα* forward: 5′-ATGAGAAGTTCCCAAATGGC-3′, and reverse: 5′-TTGTCTTTGAGATCCATGCC-3′; mouse *Vegfa* forward: 5′-CGAGGCAGCTTGAGT TAAACG-3′, and reverse: 5′-GATGATGGCGTGGTGGTGAC-3′; mouse *Il1α* forward: 5′-TGCAGTCCATAACCCATGAT-3′, and reverse: 5′-GACAAACTTCTGCCTGACGA-3′; mouse *Il1β* forward: 5′-TCAGGCAGGCAGTATCACTC-3′, and reverse: 5′-TCATCTCGGAGCCTGTAGTG-3′; mouse *Il6* forward: 5′-CTCTGGGAAATCGTGGAAAT-3′, and reverse: 5′-TCTGAAGGACTCTGGCTTTG-3′; mouse *Cxcl1* forward: 5′-TGCACCCAAACCGAAGTCAT-3′, and reverse: 5′-CTCCGTTACTTGGGGACACC-3′; mouse *Nos1* forward: 5′-TCGGGTGTCGACAATCCAAG-3′, and reverse: 5′-ATTTCTTTGGCCTGTCGGGT-3′; mouse *Nos2* forward: 5′-GTGACCATGGAGCATCCCAA-3′, and reverse: 5′-TCGAACTCCAATCTCGGTGC-3′; mouse *Nos3* forward: 5′-CTCTACCGGGACGAGGTACT-3′, and reverse: 5′-CAGGAGGTCTTGCACGTAGG-3′.

### Western blot analysis

Cell extracts were prepared using RIPA lysis buffer (150 mM sodium chloride, 1% NP-40, 0.1% SDS, 50 mM Tris, pH 7.4) containing 1 mM β-glycerophosphate, 2.5 mM sodium pyrophosphate, 1 mM sodium fluoride, 1 mM sodium orthovanadate, and protease inhibitor (Roche, Basel, Switzerland). Protein concentration was quantified using Bradford assay reagent (Bio-Rad) according to manufacturer instructions. Proteins were resolved by SDS-PAGE and then transferred to a polyvinylidene fluoride membrane (Pall Corporation, Port Washington, NY, USA). Membranes were blocked with 5% non-fat milk and incubated with the following antibodies at the indicated dilutions: anti-p21 (1:500; sc-397), anti-IκBα (1:500; sc-371), anti-p53 (1:500; sc-126, all from Santa Cruz Biotechnology), anti-p-p53 (1:500; 9286, Cell Signaling Technology), anti-ID1 (1:2,000; BCH-1-195-14, Biocheck, Foster City, CA, USA), anti-ID2 (1:500, sc-489, Santa Cruz Biotechnology), anti-ID3 (1:500, sc-490, Santa Cruz Biotechnology), anti-ID4 (1:200; ab49261, Abcam), and anti-β-actin (1:10,000; A5316, Sigma-Aldrich). Membranes were then incubated with a horseradish peroxidase-conjugated anti-IgG secondary antibody (Pierce Biotechnology, Rockford, IL, USA) and visualized using SuperSignal West Pico Chemiluminescent Substrate (Pierce Biotechnology).

### Bioinformatics data analysis

A microarray database of primary and recurrent GBM patient samples was obtained from the GEO database with accession number GSE62153 [25]. All primary GBM patients were treated with concurrent radio-chemotherapy following surgical resection. Among 43 GBM cases, we sorted and analyzed 15 paired primary and recurrent GBM cases. Additionally, samples from breast cancer patients (GSE59734 [26] and GSE101920 [27]) and colorectal cancer patients (GSE15781 [28]) treated with pre- or post-radiotherapy were obtained from the GEO database. These databases were used to determine enrichment scores (ESs) measured by single sample gene set enrichment analysis and correlation between mRNA expression of *MPO*, *CD66B*, *AIF1*, and *CD68* and *POU5F1*. For heatmap and GSEA analysis, the top 30% of recurrent GBM patients and bottom 30% of primary GBM patients were analyzed for *MPO* or *CD66B* gene expression. The TAN [29], cytokine/chemokine [29], OCT4 [30], SOX2 [30], NANOG [30], NOS [30], STAT3 [31], and NFκB gene signatures exported from the Molecular Signature Database (MSigDB) were used. The ID4 gene signature was adapted from RNA-seq data obtained from ID4-overexpressing cells (Supplementary Table S1). GSEA analysis was conducted using GSEA v17 (Broad Institute, Cambridge, MA, USA).

### Statistical analysis

Statistical analysis was performed using the two-tailed Student’s *t*-test. Values of *p* < 0.05 or *p* < 0.01 were considered statistically significant for different experiments, as indicated in the figure legends. Data are presented as means ± standard error of the mean (SEM).

## Results

### Glioblastomas analyzed after irradiation exhibit increased stem cell marker expression and tumor microenvironment changes

**Fig. 1:**
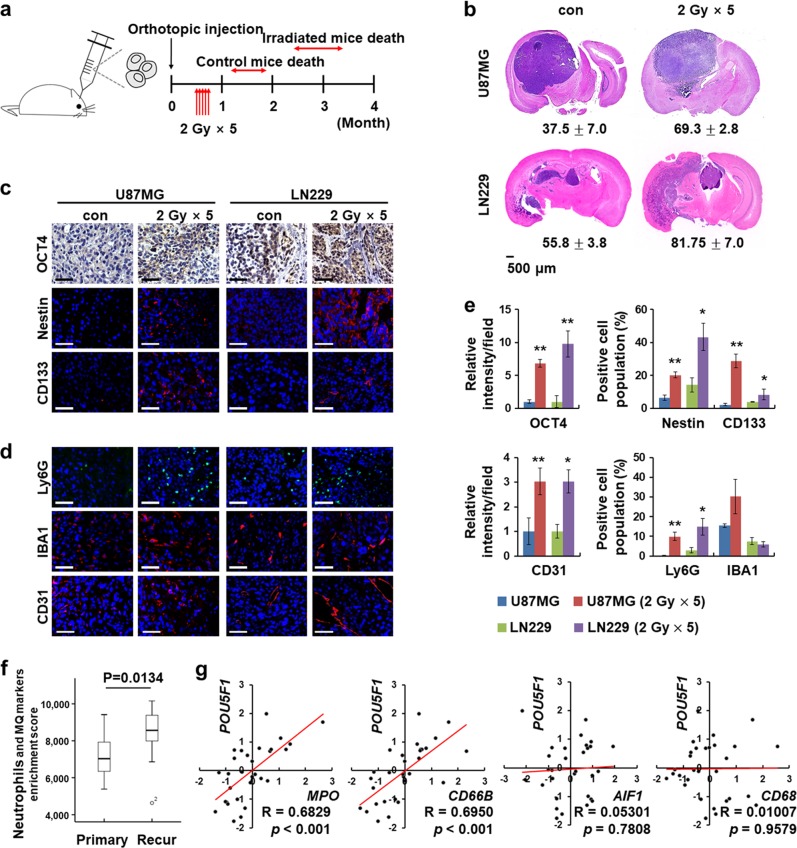
Glioblastoma grown after irradiation exhibits increased stem cell markers and microenvironmental changes. **a** Experimental scheme for the mouse model of GBM tumors treated with or without irradiation. For the irradiation of GBM mouse model, whole mouse brains were treated 5 times with 2 Gy ionizing radiation (2 Gy × 5) daily from day 21 to 25 following orthotopic injection (*n* = 4). **b** Hematoxylin–eosin (HE) stained images showing U87MG and LN229 grown tumors after fractionated irradiation. Average survival time is indicated. Scale bar represents 500μm. **c** Representative images showing the primary and grown tumors stained with several stem cell markers (OCT4, Nestin, and CD133). Scale bar represents 50 μm. **d** Representative images indicating CD31^+^ vessels, IBA1^+^ microglia, and Ly6G^+^ cells in the primary and grown tumors. Scale bar represents 50μm. **e** Quantifications of OCT4-, Nestin-, and CD133-positive cells shown in Fig. 1c and CD31-positive vessel numbers, and IBA1- and Ly6G-positive cells (*p < 0.05, **p < 0.01). **f** Box plots showing enrichment score (ES) for a gene set of neutrophil and macrophage markers of the patients with primary and recurrent GBM (*n* = 30). **g** Correlation plots of *POU5F1* mRNA levels and neutrophil markers (*MPO, CD66B*) or macrophage markers (*AIF, CD68*) mRNA level (*n* = 30). A Pearson product–moment correlation coefficient (*r*) was used to determine the linear correlation between two variables. Data in this figure are expressed as means ± standard error of the mean (SEM)

An in vivo glioblastoma irradiation model using glioblastoma cells (U87MG and LN229) was constructed to identify the cause of glioblastoma growth after radiotherapy. Mouse brains were exposed 5 times to 2 Gy irradiation (2 Gy × 5) daily at day 21 post orthotopic injection (Fig. 1a). Mice treated with whole-brain irradiation (median survival of 69.3 days in U87MG and 81.75 days in LN229) died between 3 and 5 weeks after the control mice died (median survival of 37.5 days in U87MG and 55.8 days in LN229). Hematoxylin–eosin (HE) staining images showed high infiltration of glioma cells in the mouse brain (Fig. 1b and supplementary Fig. 1). Recurrent tumors after irradiation exhibit increased stem cell-like properties, vessel formation, and inflammatory cell recruitment compared to these characteristics in primary tumors [32, 33]. Therefore, we investigated the phenotypic changes in xenograft tumors treated with or without irradiation. Similarly, expression levels of stem cell markers such as OCT4, Nestin, and CD133 (Fig. 1c, e) and CD31^+^ vessels (Fig. 1d, e) were increased in irradiated tumors compared with those in non-irradiated tumors. Surprisingly, the recruitment of Ly6G^+^ inflammatory cells increased in U87MG and LN229 irradiated tumors, and IBA1^+^ microglial cells increased only in U87MG irradiated tumors (Fig. 1d, e).

To determine whether these findings were applicable to patients diagnosed with GBM, we analyzed gene expression profiles in patients suffering from primary and recurrent GBM [25]. The recurrent GBM samples contained a higher enrichment score (ES) for neutrophil and macrophage markers compared to that of primary GBM samples (Fig. 1f). Interestingly, the expression of human neutrophil markers (*MPO* and *CD66B*) was positively correlated with the expression of *POU5F1* (OCT4, a stem cell marker), but human macrophage markers (*AIF1* and *CD68*) were not correlated with *POU5F1* (Fig. 1g). Taken together, these results suggest that neutrophils, and not macrophages, are associated with OCT4^+^ GSCs in recurrent tumors after radiotherapy.

### Irradiated glioblastoma cells trigger glioblastoma cell dedifferentiation and Ly6G^+^ inflammatory cell recruitment

**Fig. 2:**
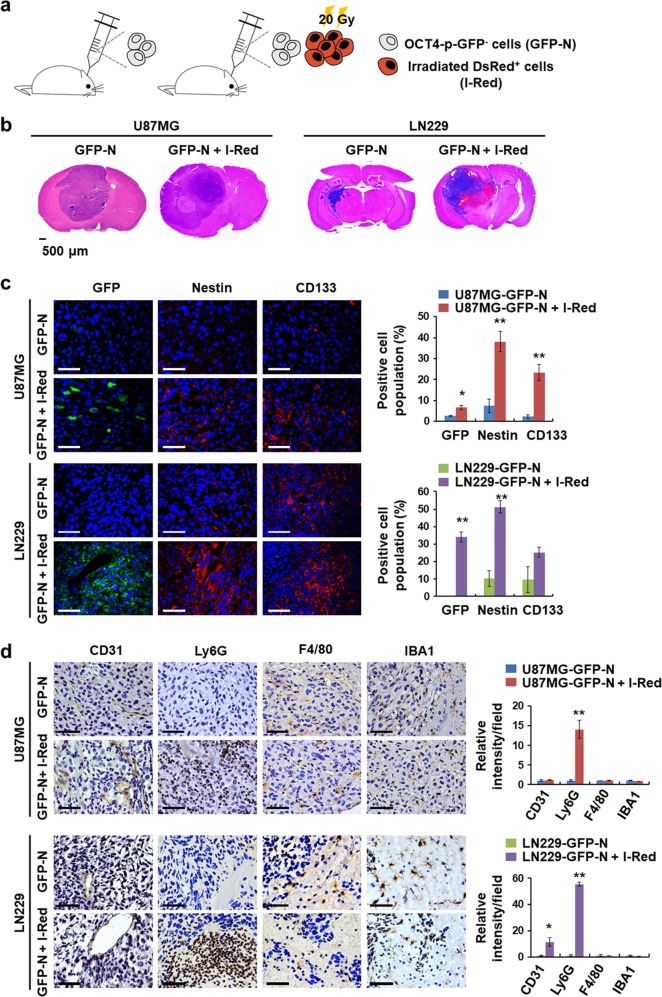
Irradiated glioblastoma cells trigger glioblastoma cell dedifferentiation and Ly6G^+^ inflammatory cell recruitment. **a** Experimental scheme for orthotopic co-injection models (*n* = 4). Mice were injected with non-irradiated glioblastoma cells (GFP-N; 1 × 10^5^) alone or with a combination of GFP-N (1 × 10^5^) and irradiated glioblastoma cells (I-Red; 2 × 10^5^). **b** HE stained images of orthotopic co-injection models. Scale bar represents 500 μm. **c** Representative immunofluorescence images of the indicated tumors stained with several stem cell markers. OCT4-, Nestin-, and CD133-positive cells were quantified (*p < 0.05, **p < 0.01). Scale bar represents 50μm. **d** Representative immunohistochemistry images of the indicated tumors stained with antibodies against CD31, Ly6G, F4/80, and IBA1. Intensity of CD31-, Ly6G-, F4/80- and IBA1-positive regions was quantified (*p < 0.05, **p < 0.01). Scale bar represents 50μm. Data in this figure are expressed as means ± SEM

We previously demonstrated that a stem cell fate-tracking system can be used to distinguish between non-stem glioblastoma cells and GSCs [34]. This system expresses the GFP gene under the control of the human *OCT4* promoter (hOCT4-p), and the GFP-positive cells exhibit characteristics of cancer stem cells [20, 34, 35]. To investigate if the irradiated glioblastoma cells promoted glioblastoma cell dedifferentiation in vivo, we established glioblastoma cell lines expressing the hOCT4-p-GFP vector and sorted GFP-negative glioblastoma cells (GFP-N). Then, we orthotopically co-injected 1 × 10^5^ U87MG-OCT4-p-GFP-negative cells (U87MG-GFP-N) or LN229-OCT4-p-GFP-negative cells (LN229-GFP-N) with irradiated 2 × 10^5^ U87MG or LN229 cells labeled with red fluorescent protein (hereafter referred to as I-Red cells) respectively, and 1 × 10^5^ U87MG-GFP-N or LN229-GFP-N cells alone, as controls (Fig. 2a). The tumors derived from co-injecting GFP-N and I-Red cells exhibited aggressive glioblastoma characteristics including hemorrhaging, invasion, and necrosis (Fig. 2b), along with high expression of stem cell markers including Nestin, CD133, and GFP (Fig. 2c). Additionally, Ly6G^+^ cells were markedly recruited in the two glioblastoma models, a finding that was different from observed changes in microglial cells and blood vessels (Fig. 2d). These results indicate that irradiated cells promote glioblastoma cell dedifferentiation and Ly6G^+^ cell recruitment.

### Irradiated glioblastoma cells enhance tumor development and progression through promoting the dedifferentiation of glioblastoma cells to GSCs

As there was no significant difference in mouse survival in the experimental model shown in Fig. 2, we could not evaluate the effect of irradiation on tumorigenicity. To demonstrate this, we injected 1 × 10^5^ GFP-N cells alone (which is not sufficient to form subcutaneous tumors), 1 × 10^5^ GFP-N cells and 5 × 10^5^ I-Red cells at a 1:5 ratios, or 5 × 10^5^ I-Red cells alone into mouse subcutaneous tissue (Supplementary Fig. 2a). Unlike mice injected with GFP-N cells or I-Red cells alone, the mice co-injected with GFP-N cells and I-Red cells developed tumors (Supplementary Fig. 2b, d), indicating that tumor formation upon co-injections was influenced by I-Red cells.

To investigate histological features of tumors derived from co-injecting GFP-N cells and I-Red cells, we examined the tumor tissues generated by injecting 1 × 10^6^ GFP-N cells as a control. Similar to the results of the orthotopic tumor model, I-Red cells promoted aggressive glioblastoma formation, which is characterized by necrosis, hemorrhaging, and inflammatory cell infiltration (Supplementary Fig. 2c, e). Additionally, in the tumor tissues generated by co-injection the number of dedifferentiated cells expressing OCT4, GFP (Supplementary Fig. 2f), and Nestin (Supplementary Fig. 2g) was increased, whereas the number of cells expressing the differentiated astrocyte marker GFAP was decreased (Supplementary Fig. 2g). Vessel formation and infiltration by Ly6G^+^ cells and macrophages were all significantly promoted in tumors derived from the co-injection model (Supplementary Fig. 2h). Taken together, these results indicate that irradiated glioblastoma cells also promote aggressive tumor development and progression.

To confirm whether these cells were still present in the tumor tissues, I-Red cells were stained with anti-RFP antibodies in tumors formed by co-injecting GFP-N cells and I-Red cells. There were no RFP-positive cells in either the orthotopic model (Supplementary Fig. 3a) or the subcutaneous model (Supplementary Fig. 3b). These results suggest that irradiated cells may affect the non-irradiated cells and host microenvironment in the early stages of tumorigenesis and progression, and that these irradiated cells may then be removed by inflammatory cells [36]. It has been reported that irradiation increases stem cell populations in breast cancer [37]. We examined sphere-forming ability and stem cell marker expression in the irradiated U87MG and LN229 cells. As a result, the irradiated cells did not show increased expression of stem cell markers (Supplementary Fig. 3c). Therefore, we conclude that I-Red cells themselves do not acquire stem cell characteristics and do not form tumor bulk in vivo.

### Tumor-derived OCT4-p-GFP-positive cells possess stem cell-like properties and express ID4, a master regulator of GSCs

**Fig. 3:**
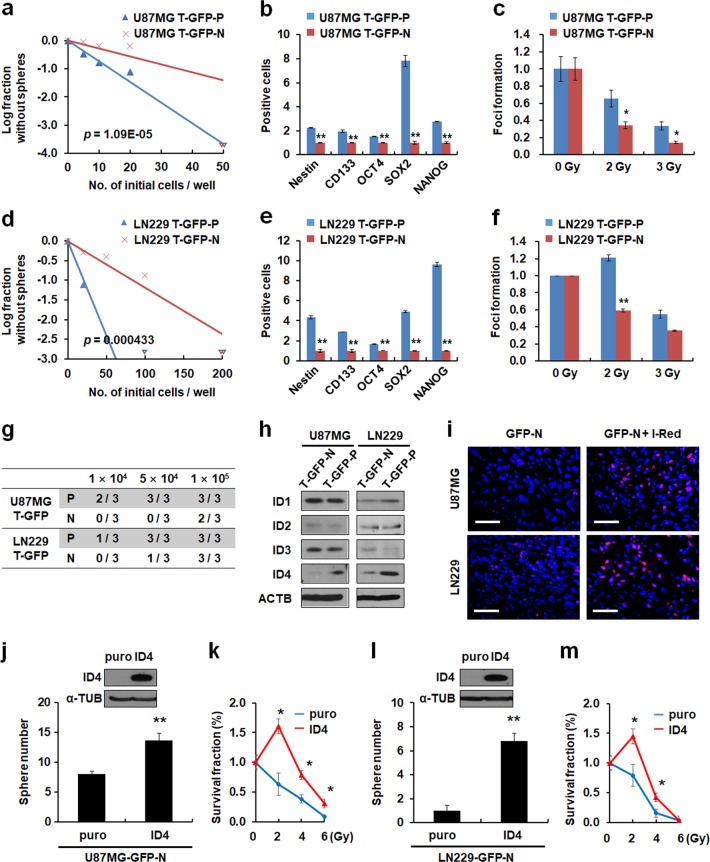
Tumor-derived GFP-P cells exhibit stem cell-like properties and ID4 expression. **a**, **d** In vitro limiting dilution assay showing stem cell sphere-forming frequency of the T-OCT4-p-GFP^+^ (T-GFP-P) and T-OCT4-p-GFP^-^ (T-GFP-N) cells derived from a subcutaneous co-injection model (Supplementary Fig. 2a). **b**, **e** FACS analysis comparing the expression of several stem cell markers (Nestin, CD133, OCT4, SOX2, and NANOG) in T-GFP-P and T-GFP-N cells (*p < 0.05, **p < 0.01). **c**, **f** Colony-formation assay demonstrating the radioresistance of T-GFP-P and T-GFP-N cells on day 14 after 0, 2, or 3 Gy irradiation (*p < 0.05, **p < 0.01). **g** Tumor-initiating ability of T-GFP-P and T-GFP-N cells. **h** Western blot analysis showing ID family expression in T-GFP-P and T-GFP-N cells. **i** Representative images indicating ID4-positive cells (red) within the tumors. ID4-positive cells were quantified (*p < 0.05, **p < 0.01). Scale bar represents 50 μm. **j** Western blot analysis showing ID4 expression (upper) and sphere-forming assay (bottom) of U87MG-GFP-N-puro and -ID4 cells. **k** MTS assay for detecting radioresistance of U87MG-GFP-N-puro and -ID4 cells. Cell survival was detected 3 days post irradiation. **l** Western blot analysis showing ID4 expression (upper) and sphere-forming assay results (bottom) of LN229-GFP-N-puro and -ID4 cells. **m** MTS assay for detecting radioresistance of LN229-GFP-N-puro and -ID4 cells. Cell survival was detected 3 days post irradiation. Data in this figure are expressed as means ± SEM

To characterize OCT4-p-GFP-positive (GFP-P) cells derived from GFP-N cells, we sorted OCT4-p-GFP-positive cells (T-GFP-P) and OCT4-p-GFP-negative cells (T-GFP-N) from the subcutaneous tumors derived from co-injection of GFP-N cells and I-Red cells. T-GFP-P cells exhibited increased sphere-forming ability (Fig. 3a, d), stem cell marker expression (Fig. 3b, e), radiation resistance (Fig. 3c, f), and tumor-initiating capacity (Fig. 3g) as compared to T-GFP-N cells. Additionally, we also confirmed stem cell-like properties of T-GFP-P cells derived from the orthotopic co-injection model (Supplementary Fig. 4), suggesting that T-GFP-P cells exhibited higher stem cell properties than T-GFP-N cells.

Previously, we reported that inhibitor of differentiation (ID) family members regulate stem cell-like properties of GSCs and dedifferentiation of glioblastoma cells into GSCs [38–40]. Therefore, we examined the expression level of four ID proteins and found that ID4 protein expression was significantly elevated in T-GFP-P cells (Fig. 3h) and in tumor tissues derived from co-injection models (Fig. 3i). To investigate if the acquisition of stem cell-like properties is directly stimulated by ID4, we overexpressed ID4 in GFP-N cells (Fig. 3j, l) and found that ID4 enhanced sphere-forming ability (Fig. 3j, l) and radiation resistance (Fig. 3k, m). These results suggest that dedifferentiated T-GFP-P cells can partially acquire GSC properties through ID4 expression.

### Irradiated glioblastoma cells directly increase vessel formation and inflammatory cell recruitment in vitro

To determine whether glioblastoma cells were directly dedifferentiated by irradiated cells, we co-cultured GFP-N cells with I-Red in vitro. GFP expression was not significantly increased in GFP-N cells (Supplementary Fig. 5a). Next, we performed in vitro tubule formation assays on human retinal endothelial cells using conditioned medium (CM) from I-Red cells or non-irradiated control U87MG and LN229 cells (N-Red). Tubule formation was significantly increased upon exposure to CM taken from I-Red cells (Supplementary Fig. 5b). We also performed in vitro transwell invasion assays using mouse microglial cells (BV2) and in vitro transwell migration assays using differentiated monocyte cells (dHL-60; neutrophil lineage). The recruitment of BV2 and dHL-60 cells was significantly increased upon exposure to the CM from I-Red cells compared to that observed after exposure to the CM from N-Red cells (Supplementary Fig. 5c, d). Therefore, our results suggest that irradiated cells facilitate angiogenesis and inflammatory cell recruitment rather than dedifferentiation of glioblastoma cells into GSCs.

### Irradiated glioblastoma cells promote changes to the tumor microenvironment via NFκB signaling in vitro and in vivo

**Fig. 4:**
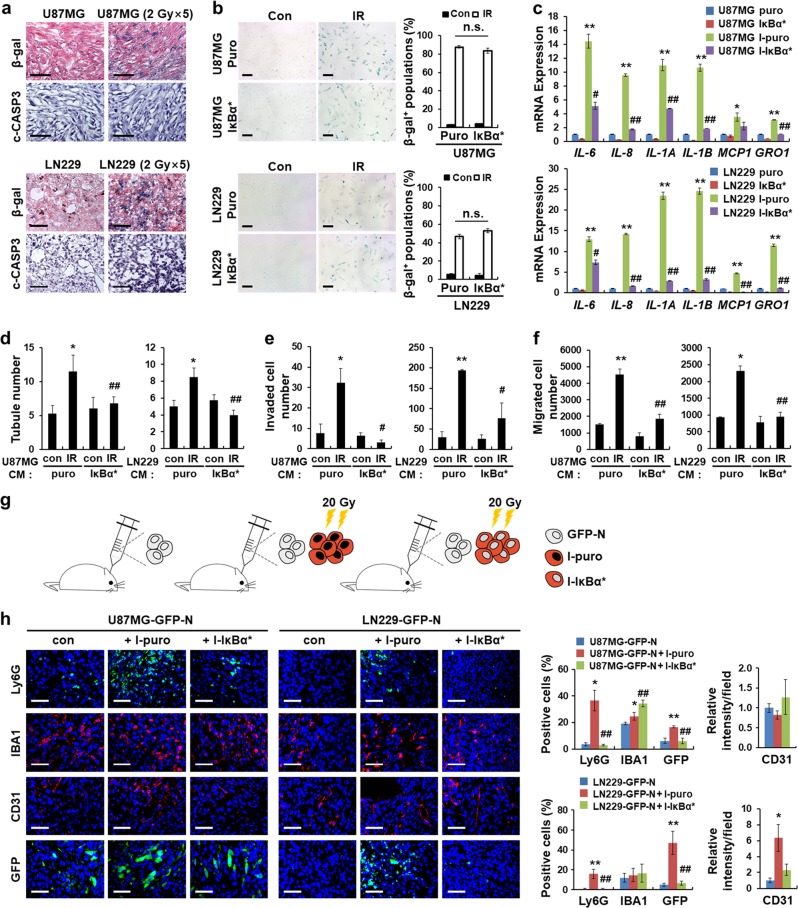
Irradiated glioblastoma cells promote tumor microenvironment changes via NFκB signaling in vitro and in vivo. **a** Representative images showing SA-β-gal- and c-CASP3-positive cells in U87MG and LN229 xenograft models after fractionated irradiation (2 Gy × 5). Scale bar represents 50 μm. **b** The SA-β-gal staining assay of U87MG and LN229 cells overexpressing IκBα* on day 3 after irradiation with 20Gy. Representative images (left) and quantification of the SA-β-gal-positive glioblastoma cells (right, n.s.: not significant). Scale bar represents 50μm. **c** qRT-PCR assay showing mRNA levels of SASP genes in U87MG and LN229 cells overexpressing IκBα* on day 3 after irradiation with 20 Gy (*p < 0.05, **p < 0.01 between puro and I-puro; ^#^*p* < 0.05, ^##^*p* < 0.01 between I-puro and I-IkBα*). **d** In vitro tubule formation assay of HRECs incubated with CM obtained from I-puro cells or I-IκBα* cells. Quantification of the tube numbers (*p < 0.05, **p < 0.01 between control-puro and IR-puro; ^#^*p* < 0.01 between IR-puro and IR-IkBα*). **e** Transwell invasion assay of BV2 microglial cells in the upper chamber with CM from I-puro cells or I-IκBα* cells in the bottom chamber. Quantification of the total number of invaded cells (*p < 0.05, **p < 0.01 between control-puro and IR-puro; ^##^*p* < 0.01 between IR-puro and IR-IkBα*). **f** Transwell migration assay of dHL-60 cells in the upper chamber with CM from I-puro cells or I- IκBα* cells in the bottom chamber. (*p < 0.05, **p < 0.01 between control-puro and IR-puro; ^##^*p* < 0.01 between IR-puro and IR-IkBα*). **g** Experimental scheme for the orthotopic co-injection models (*n* = 4). Mice were injected with GFP-N alone (1 × 10^5^), GFP-N (1 × 10^5^) and I-puro (2 × 10^5^), or I-IκBα* alone (2 × 10^5^). **h** Representative immunofluorescence images of the indicated tumors stained with Ly6G, IBA1, CD31, and GFP. Ly6G-, IBA1-, CD31-, and GFP-positive cells were quantified (*p < 0.05, **p < 0.01 between GFP-N and GFP-N + I-puro; ^#^*p* *<* 0.05, ^##^*p* *<* 0.01 between GFP-N + I-puro and GFP-N + I-IkBα*). Scale bar represents 50μm. Data in this figure are expressed as means ± SEM

Our previous study demonstrated that irradiation induces cellular senescence and the SASP through NFκB signaling in vitro [18]. To examine the fate of irradiated cells in vivo, we subcutaneously co-injected U87MG or LN229 cells in combination with I-Red cells into mice. We identified RFP, SA-β-gal (an indicator of senescent cells), and c-CASP3 (an indicator of apoptotic cells) by staining tumor tissues harvested at the indicated days. The results showed that most RFP^+^ I-Red cells express SA-β-gal, but not c-CASP3 (Supplementary Fig. 6). Additionally, we found that in mouse xenograft models irradiation increased the number of SA-β-gal^+^ cells, but not the number of c-CASP3^+^ cells (Fig. 4a). Therefore, these results indicate that irradiated glioblastoma cells exist in a senescent state in vivo.

The SASP includes a variety of cytokines and chemokines that regulate tumor cell proliferation, invasion, migration, and inflammatory cell recruitment [36, 41]. We wondered whether the SASP from irradiation-induced senescent cells regulates the tumor microenvironment. To confirm this, we suppressed NFκB signaling by expressing an IκBα super repressor (IκBα*) lacking phosphorylation sites that induce proteasomal degradation. After irradiation, U87MG and LN229 cells expressing IκBα* retained their IκBα protein level but exhibited increased levels of p-p53, p53, and p21^Cip1^ protein (Supplementary Fig. 7) and there were also elevated levels of SA-β-gal-positive cells (Fig. 4b). After irradiation, however, the expression of SASP-related mRNAs in these cells markedly decreased (Fig. 4c). These results indicate that NFκB signaling is not important for inducing glioblastoma cell senescence after irradiation, but this signaling is crucial for inducing SASP.

Next, using CM obtained from the U87MG-puro or U87MG-IκBα* and LN229-puro or LN229-IκBα* cell lines irradiated with 20 Gy (which are designated as I-puro or I-IκBα*, respectively), we performed in vitro tubule formation assays using HREC cells and transwell invasion and migration assays using inflammatory cells. As a result, tubule formation of HRECs (Fig. 4d) and invasion (Fig. 4e) and migration (Fig. 4f) of inflammatory cells increased upon exposure to the CM taken from I-puro cells than upon exposure to the CM taken from I-IκBα* cells. To further investigate the effects of NFκB signaling blockade on tumor microenvironment alterations caused by irradiation, we performed orthotopic co-injection of GFP-N cells with I-puro cells or I-IκBα* cells (Fig. 4g). GFP^+^ and Ly6G^+^ cells were affected by NFκB signaling inhibition in two orthotopic xenograft models (Fig. 4h). These results indicate that NFκB signaling plays a critical role in inducing the radiation-induced SASP, which in turn promotes changes in the tumor microenvironment.

### Infiltrated Ly6G^+^ inflammatory cells promote dedifferentiation of glioblastoma cells via the NO-ID4 axis

**Fig. 5:**
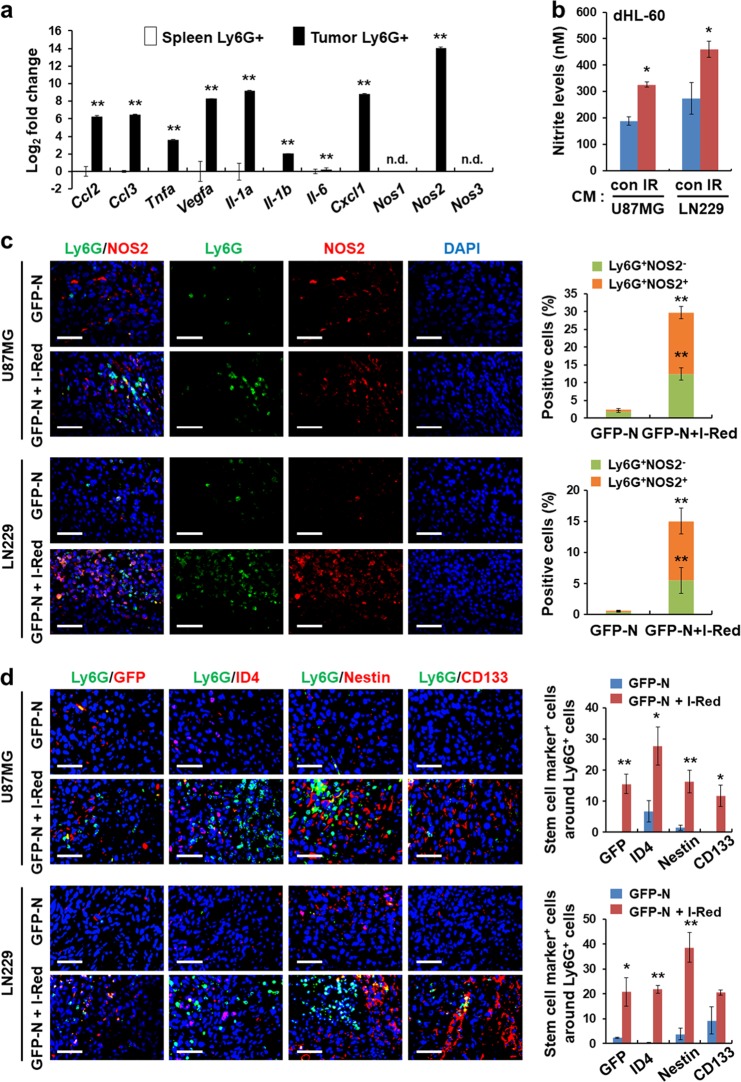
Infiltrated Ly6G^+^ inflammatory cells promote dedifferentiation of glioblastoma cells to GSCs via the NO-ID4 axis. **a** qRT-PCR assay showing mRNA levels of pro-inflammatory cytokines and chemokines (*Ccl2*, *Ccl3*, *Tnf-a*, *Vegfa*, *Il-1a*, *Il-1b*, *Il-6*, *Cxcl1*, *Nos1*, *Nos2*, and *Nos3*) in Ly6G^+^ cells isolated from normal spleen and tumor tissues (*p < 0.05, **p < 0.01; n.d. not detectable). **b** Quantification of nitrite levels secreted by I-Red cells derived from U87MG and LN229 cells. **c** Representative immunofluorescence images showing Ly6G (green)/NOS2 (red) double-positive cells. Ly6G^+^NOS2^-^ and Ly6G^+^NOS2^+^ cells were quantified (**p < 0.01). **d** Representative images showing GFP-, ID4-, Nestin-, and CD133-positive GSCs (red) located in close proximity to the Ly6G^+^ cells (green). The stem cell marker-positive cells located in close proximity to the Ly6G-positive cells (≤100μm diameter regions) were quantified (*p < 0.05, **p < 0.01). Data in this figure are expressed as means ± SEM

Ly6G^+^ cells significantly infiltrated into tumors after exposure to fractionated irradiation or tumors derived from co-injecting irradiated and non-irradiated glioblastoma cells. These Ly6G^+^ cells may be an important factor in glioblastoma cell dedifferentiation after radiotherapy, as their expression also correlates with the expression of *POU5F1* observed in recurrent GBM patients after radiotherapy (Fig. 1g). To confirm this, we isolated Ly6G^+^ cells from tumors obtained by co-injecting GFP-N cells and I-Red cells, and we characterized them. As the number of infiltrated Ly6G^+^ cells was low in the control tumors, we used Ly6G^+^ cells isolated from normal spleen as controls. We found that tumor-derived Ly6G^+^ cells express many types of cytokines and chemokines (Fig. 5a). In particular, *NOS2* mRNA was significantly increased, but *NOS1* and *NOS3* mRNA was not detected (Fig. 5a).

Previously, we reported that NO is important for inducing ID4 expression and dedifferentiation through crosstalk between GSCs and endothelial cells [13, 34]. As T-GFP-P cells exhibited high ID4 expression (Fig. 3h), we wondered if the NOS-ID4 axis was involved in Ly6G^+^ cells-induced dedifferentiation. To determine this, we measured nitrite levels, which reflect NOS activity, in vitro. Nitrite secretion was increased in dHL-60 cells cultured with CM derived from irradiated cells compared to secretion observed in cells grown in the CM derived from non-irradiated cells (Fig. 5b). Double-positive (Ly6G^+^NOS2^+^) cells were significantly increased in tumors derived from co-injecting GFP-N and I-Red cells (Fig. 5c). These results suggest that the irradiated cells promote NOS activity and NO secretion in infiltrated Ly6G^+^ cells in tumor tissues. To analyze the relationship between Ly6G^+^ cells and dedifferentiated cells in vivo, we subcutaneously injected non-irradiated cells alone or non-irradiated cells in combination with I-Red cells (Supplementary Fig. 8a), and then performed two experimental analyses. First, we identified the location of these cells within the tumors. Dedifferentiated GFP-P cells and other stem cell marker-positive cells were located near Ly6G^+^ cells in U87MG and LN229 tumor models generated by co-injection of non-irradiated cells and I-Red cells (Fig. 5d). Second, we performed FACS analysis using single cells obtained from the tumors at an early time point (days 15 and 30 post-injection). The recruitment of F4/80^+^ cells was either unchanged in U87MG tumors or slightly increased in the LN229 tumor, but the increase in recruitment of Ly6G^+^ cells was significantly correlated with accumulation of Nestin^+^ and CD133^+^ cells observed in the co-injection tumor models of U87MG and LN229 cells (Supplementary Fig. 8b, c). Together, these results indicate that NO secreted by Ly6G^+^ cells promote the NOS-ID4 signaling axis that converts glioblastoma cells to GSCs.

### Inhibiting Ly6G^+^ inflammatory cells delays tumor growth after radiotherapy

**Fig. 6:**
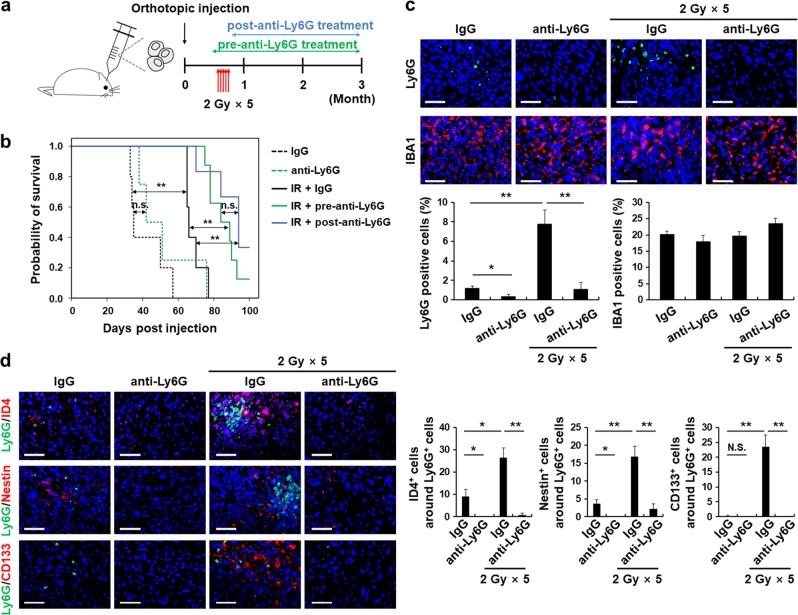
Ly6G^+^ inflammatory cell inhibition delays tumor growth after irradiation. **a** Experimental scheme for the recurrent GBM model treated with either control IgG or anti-Ly6G. The mice were treated 5 times with 2 Gy ionizing radiation (2 Gy × 5) from day 21 to 25 after orthotopic injection (*n* = 5). In one group of mice (pre-anti-Ly6G treatment), IgG or anti-Ly6G antibody was injected at a dose of 300 μg on day 19 prior to irradiation and then at 100 μg after irradiation. In a second group (post-anti-Ly6G treatment), 100 μg of antibodies were injected every 72 h from day 26 until the final treatment. **b** Survival rate of U87MG xenograft mice injected with either IgG or anti-Ly6G before and after exposure to irradiation (0 Gy or 2 Gy × 5; *p < 0.05, **p < 0.01; n.s. not significant). **c** Representative immunofluorescence images and quantification of Ly6G^+^ and IBA1^+^ cells. (*p < 0.05, **p < 0.01). **d** Representative images showing ID4^+^, Nestin^+^, and CD133^+^ GSCs (red) located in close proximity to the Ly6G^+^ cells (green). The stem cell marker-positive cells located in close proximity to the Ly6G^+^ cells (≤100 μm diameter regions) were quantified (*p < 0.05, **p < 0.01). Data in this figure are expressed as means ± SEM

To further investigate the significance of infiltrated Ly6G^+^ cells in tumor growth, Ly6G^+^ cells were neutralized using a Ly6G-specific antibody in mice. Treatment with the isotype control (IgG) or anti-Ly6G was initiated before and after mouse brain irradiation (pre- and post-anti-Ly6G treatment, respectively) (Fig. 6a). Depletion of Ly6G^+^ cells significantly increased the survival rate of irradiated mice, but did not affect the survival rate of non-irradiated mice (Fig. 6b). There was no significant difference in mouse survival promoted by pre- and post-anti-Ly6G treatment (Fig. 6b). We confirmed that anti-Ly6G treatment depleted Ly6G^+^ cells in the blood (Supplementary Fig. 9a), spleen (Supplementary Fig. 9b), and tumors (Fig. 6c), but treatment did not affect IBA1^+^ macrophages (Supplementary Fig. 9b; and Fig. 6c). As the non-irradiated mice possessed fewer Ly6G^+^ cells (less than 2%) compared to irradiated mice (Fig. 6c), it is likely that anti-Ly6G treatment would have little effect on the prevention of tumor growth (Fig. 6b). Also, ID4^+^, CD133^+^ or Nestin^+^ cells in close proximity to the Ly6G^+^ cells were dramatically decreased in the tumors of irradiated mice (Fig. 6d). Taken together, our results indicate that Ly6G^+^ cells play important roles in tumor growth after radiotherapy.

### Neutrophils correlate with TAN and dedifferentiation gene sets in patients diagnosed with recurrent GBM

**Fig. 7:**
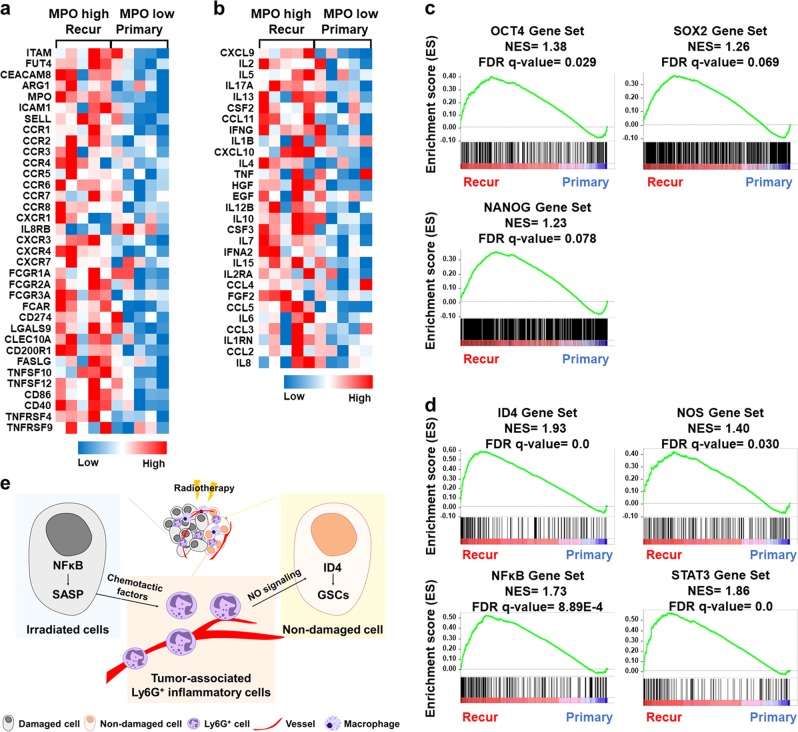
Neutrophils correlate with TAN and dedifferentiation gene sets in recurrent GBM patient samples. **a**, **b** Heatmap of TAN (**a**) and cytokine/chemokine (**b**) genes observed in recurrent GBM patient samples with *MPO* high vs. primary GBM patient samples with *MPO* low. **c**, **d** GSEA data showing the enrichment of OCT4, SOX2, and NANOG gene sets (**c**) and ID4, NOS, NFκB, and STAT3 gene sets (**d**) in recurrent GBM patient samples with *MPO* high. **e** A schematic diagram indicating the mechanism of GBM recurrence after radiotherapy. Ly6G^+^ inflammatory cells recruited by radiation-induced SASP promote GBM recurrence by inducing the dedifferentiation of glioma cells to GSCs via the NOS-ID4 signaling axis

To assess if neutrophils and dedifferentiation signatures were associated in patients with recurrent GBM, we examined the RNA sequencing datasets of patients with primary and recurrent GBM [25]. Recurrent GBM samples expressing high *MPO* or *CD66B* gene levels were positively correlated with TAN markers (Fig. 7a; and Supplementary Fig. 10a) and cytokine/chemokine gene expression (Fig. 7b; and Supplementary Fig. 10b) [29]. These data suggest that infiltrated neutrophils in patients with recurrent GBM exhibit the TAN phenotype. Additionally, gene set enrichment analysis (GSEA) demonstrated that the gene sets related to OCT4, SOX2, and NANOG were highly enriched in recurrent GBM samples (Fig. 7c; and Supplementary Fig. 10c). The GSEA also indicated that the gene sets related to ID4, NOS, NFκB, and STAT3 were highly enriched in recurrent GBM samples (Fig. 7d; and Supplementary Fig. 10d), indicating that the increased expression of cytokine/chemokine genes in patients with recurrent GBM is associated with NFκB and STAT3 signaling. We also analyzed the ES of neutrophils and MQ markers, stemness signaling, and inflammation signaling in other solid cancer patient samples pre- or post radiotherapy [26–28]. Although the ES of neutrophils and MQ markers was not significantly increased due to small sample sizes, GSEA showed several gene sets were enriched in patients post-radiotherapy compared to pre-radiotherapy (Supplementary Fig. 11). Since most GBM patients receive concurrent chemoradiotherapy, we also confirmed the effects of TMZ treatment alone or in combination with ionizing radiation on glioblastoma cells. Similar to radiation treatment alone, a single treatment of TMZ or a combined treatment of TMZ and radiation on glioblastoma cells promoted cellular senescence (Supplementary Fig. 12a, c) rather than apoptosis (Supplementary Fig. 12b, d).

In conclusion, these results suggest that infiltrated Ly6G^+^ inflammatory cells displaying the TAN phenotype increase the expression of cytokines and chemokines and promote dedifferentiation signaling, which is closely related to the initiation of GBM recurrence (Fig. 7e). Therefore, our results provide a novel insight into tumor recurrence after radiotherapy and provide a basis for new therapeutic strategies for GBM.

## Discussion

Despite concurrent chemoradiotherapy, most GBM tumors recur in proximity to the radiation field. The effect of radiotherapy on GBM recurrence, however, remains unclear. Here, we demonstrated that ionizing radiation induces glioblastoma cell senescence and the SASP, which in turn promotes Ly6G^+^ inflammatory cell recruitment with TAN phenotype into tumor tissues and changes to the tumor microenvironment.

Although G-MDSCs and TANs express Ly6G protein and play an important role in tumor progression, transcriptomic analysis suggests that G-MDSCs are more closely related to naïve neutrophils than TANs are and that cytokine secretion is increased in G-MDSCs to a greater degree than in TANs [42]. Thus, despite the fact that G-MDSCs can differentiate into TANs [43], our results suggest that infiltrated Ly6G^+^ cells in recurrent tumors are similar to neutrophils acting as protumorigenic TANs expressing CCL2, CCL3, and CXCL1 [42].

Bone marrow-derived cells (BMDCs), which are CD11b-positive myelomonocytes, are important in GBM regrowth after irradiation through promoting vascular restoration [33]. We also confirmed the increased number of macrophages and blood vessels using a subcutaneous co-injection model. It was, however, difficult to confirm the increase in macrophages in our orthotopic tumor irradiation model generated by co-injecting non-irradiated and irradiated glioblastoma cells. This is because the orthotopic injections of U87MG or LN229 cells form tumors containing high numbers of macrophages (8–15%) compared to those formed by subcutaneous injections (less than 3%). The subcutaneous co-injection model increases macrophage populations (9–14%) to percentages similar to the percentage of macrophages in tumors derived from orthotopic injection of U87MG or LN229 cells. Although the numbers of recruited macrophages did not change, these cells showed activated microglial morphology with contracted branches and an amoeboid shape (Fig. 2d) [44]. Therefore, increased microglia activation may be involved in tumor progression and recurrence in the orthotopic injection model. Although we have identified the role of Ly6G^+^ cells and macrophages in xenograft tumors using immune-compromised nude mice, Ly6G^+^ cells can suppress the cytotoxic effects of T cells [6, 45]. Therefore, further studies to define the relationship between Ly6G^+^ cells and the adaptive immune system should be performed using syngeneic GBM mouse models.

## Supplementary information

Supplementary Figures
