# Supplementary material for: Ly6G+ inflammatory cells enable the conversion of cancer cells to cancer stem cells in an irradiated glioblastoma model
Source: Cell Death Differ. 2019 Feb 25;26(10):2139–56. doi: 10.1038/s41418-019-0282-0 (PMC6748155; doi:10.1038/s41418-019-0282-0)
Supplement: Supplementary file 1 — Supplementary Figures [file 41418_2019_282_MOESM1_ESM.pdf]

ONLINE SUPPLEMENTARY INFORMATION

Ly6G$^+$ inflammatory cells enable the conversion of cancer cells to cancer stem cells in an irradiated glioblastoma model

Hee-Young Jeon[1,2], Seok Won Ham[1,2], Jun-Kyum Kim[1,2], Xiong Jin[1,2], Seon Yong Lee[1], Yong Jae Shin[4,5], Chang-Yong Choi[1,2], Jason K. Sa[5], Se Hoon Kim[7], Taehoon Chun[1,2], Xun Jin[8,9], Do-Hyun Nam[4,5,6] and Hyunggee Kim[1,2,3,*]

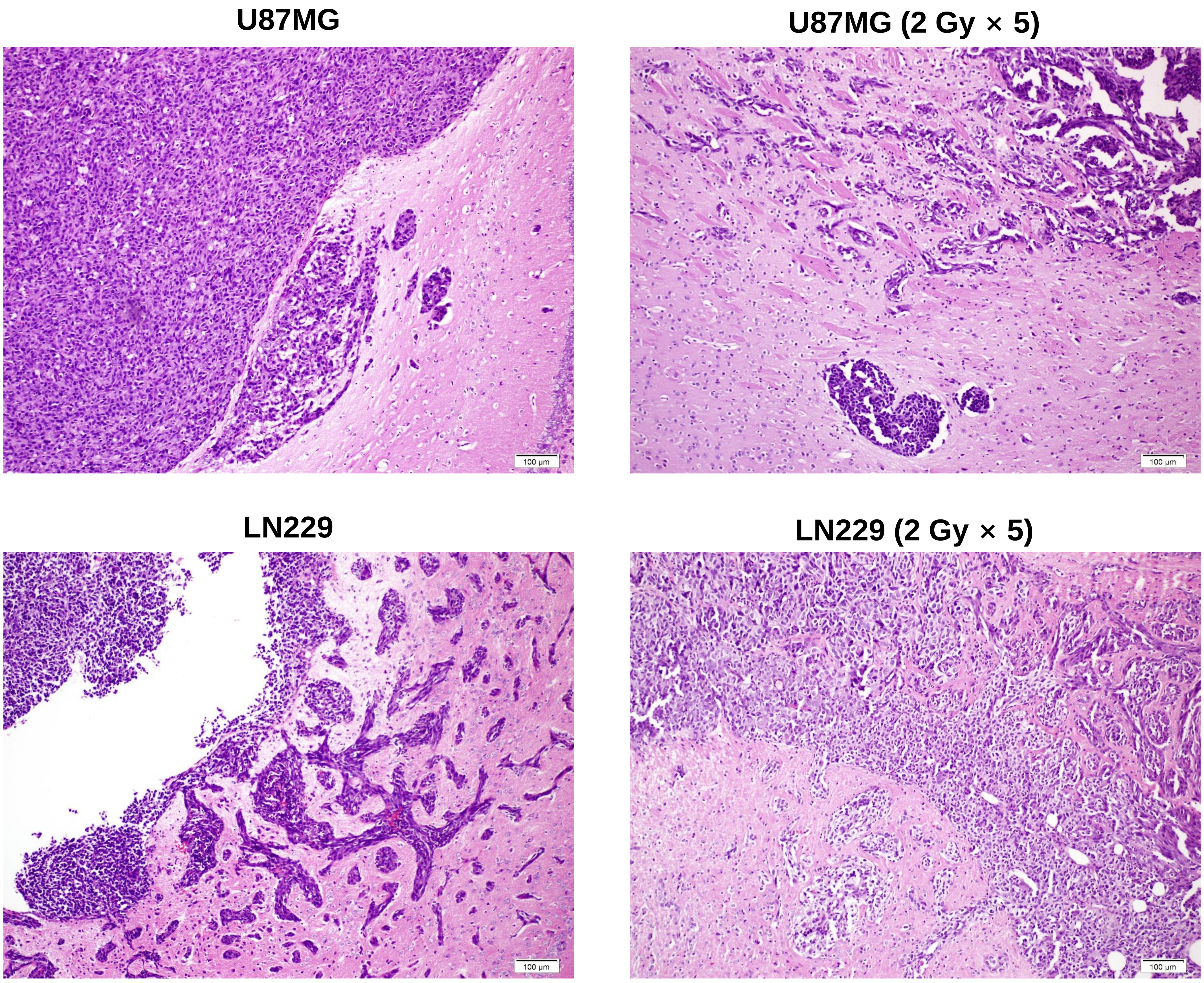

Supplementary Fig. 1 Histological features of U87MG and LN229 xenograft tumors.

High-magnification images of HE staining data shown in Fig. 1b represent highly infiltrative glioma cells. Scale bar represents 100 μm.

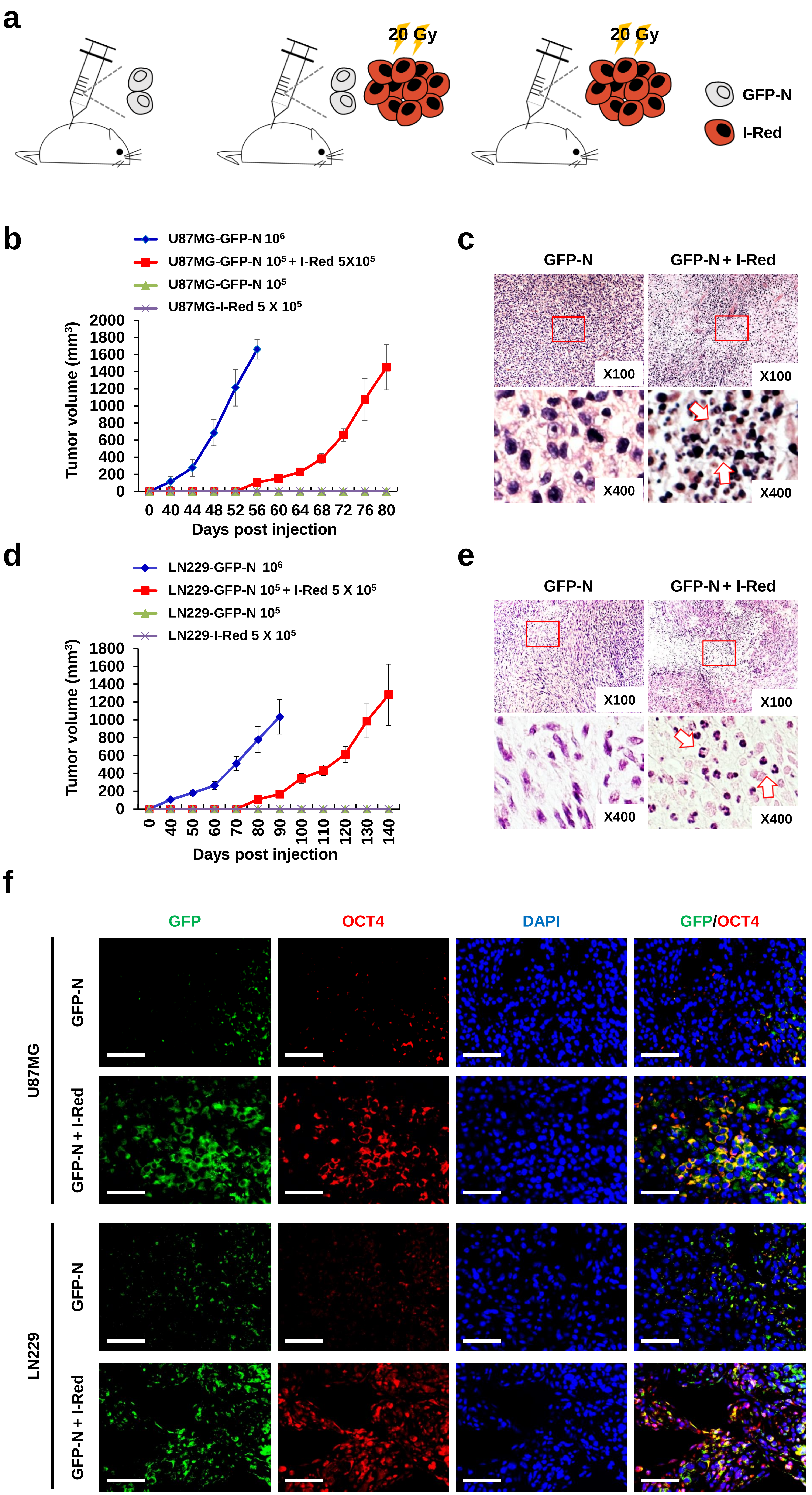
a
20 Gy
20 Gy
GFP-N
I-Red
b
U87MG-GFP-N 10^6
U87MG-GFP-N 10^5 + I-Red 5X10^5
U87MG-GFP-N 10^5
U87MG-I-Red 5 X 10^5
Tumor volume (mm^3)
Days post injection
c
GFP-N
GFP-N + I-Red
X100
X400
d
LN229-GFP-N 10^6
LN229-GFP-N 10^5 + I-Red 5 X 10^5
LN229-GFP-N 10^5
LN229-I-Red 5 X 10^5
Tumor volume (mm^3)
Days post injection
e
GFP-N
GFP-N + I-Red
X100
X400
f
GFP
OCT4
DAPI
GFP/OCT4
U87MG
LN229
GFP-N
GFP-N + I-Red

(Continued)

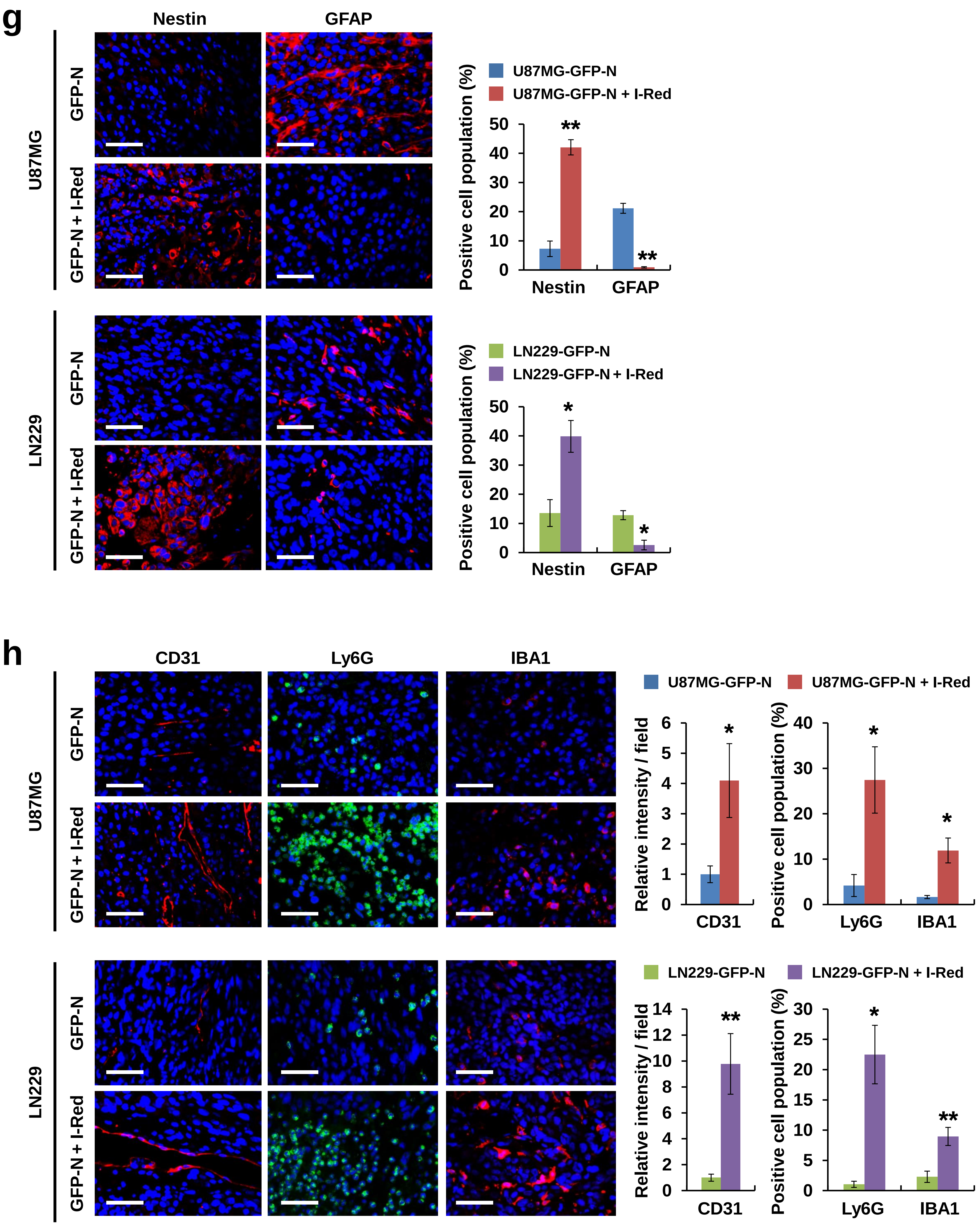

See the next page for figure legend

Supplementary Fig. 2 Irradiated glioma cells enhance tumor development and progression by promoting the dedifferentiation of glioma cells to GSCs.

(a) Experimental scheme for subcutaneous co-injection models (n = 4). Mice were injected with GFP-N (1×10^6 or 1×10^5) alone, GFP-N (1×10^5) and I-Red (5×10^5), or I-Red (5×10^5) alone. The group of GFP-N (1×10^6) alone was used as a positive control as the group of GFP-N (1×10^5) alone did not form tumors.

(b, d) The mouse tumor volumes of the indicated groups.

(c, e) HE staining images showing heterogeneous cell populations and necrotic regions in the U87MG (c) and LN229 (e) tumors derived from co-injecting GFP-N and I-Red cells compared to those derived from injecting GFP-N alone.

(f) Representative images showing GFP (green) and OCT4 (red) double-positive cells in the U87MG and LN229 tumors as indicated. Scale bar represents 50 μm.

(g) Representative images showing Nestin and GFAP in the U87MG and LN229 tumors as indicated. Nestin- and GFAP-positive cells were quantified (*$p < 0.05$, **$p < 0.01$; n = 3). Scale bar represents 50 μm.

(h) Representative images showing CD31, Ly6G, and IBA1 in the U87MG and LN229 tumors as indicated. CD31-, Ly6G-, and IBA1-positive cells were quantified (*$p < 0.05$, **$p < 0.01$; n = 3). Scale bar represents 50 μm.

Data in this figure are expressed as means ± SEM.

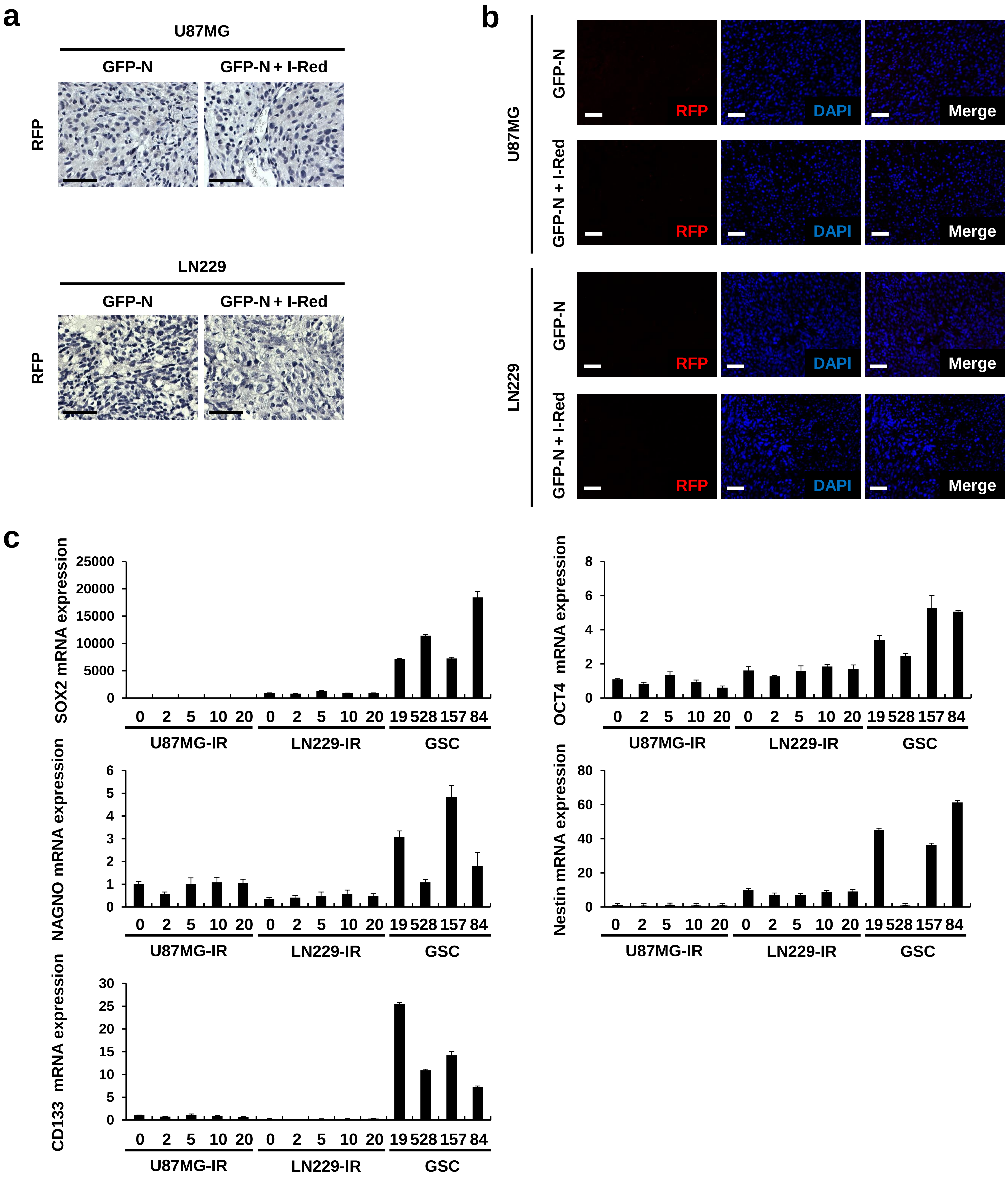

See the next page for figure legend

Supplementary Fig. 3 Tumors derived from co-injection models show a loss of I-Red cells.

(a) Representative immunohistochemistry images stained with anti-RFP primary antibody to confirm the I-Red cells. There were no RFP-positive cells in the orthotopic tumors derived from co-injecting GFP-N and I-Red cells. Scale bar represents 50 μm.

(b) Representative immunofluorescence images stained with anti-RFP primary antibody to confirm the I-Red cells. There were no RFP-positive cells in the subcutaneous tumors derived from co-injecting GFP-N and I-Red cells. Scale bar represents 50 μm.

(c) qRT-PCR analysis of stem cell-like markers in U87MG and LN229 cells following irradiation (0, 2, 5, 10, and 20 Gy) and 4 GSCs used as positive controls. Data are expressed as means ± SEM.

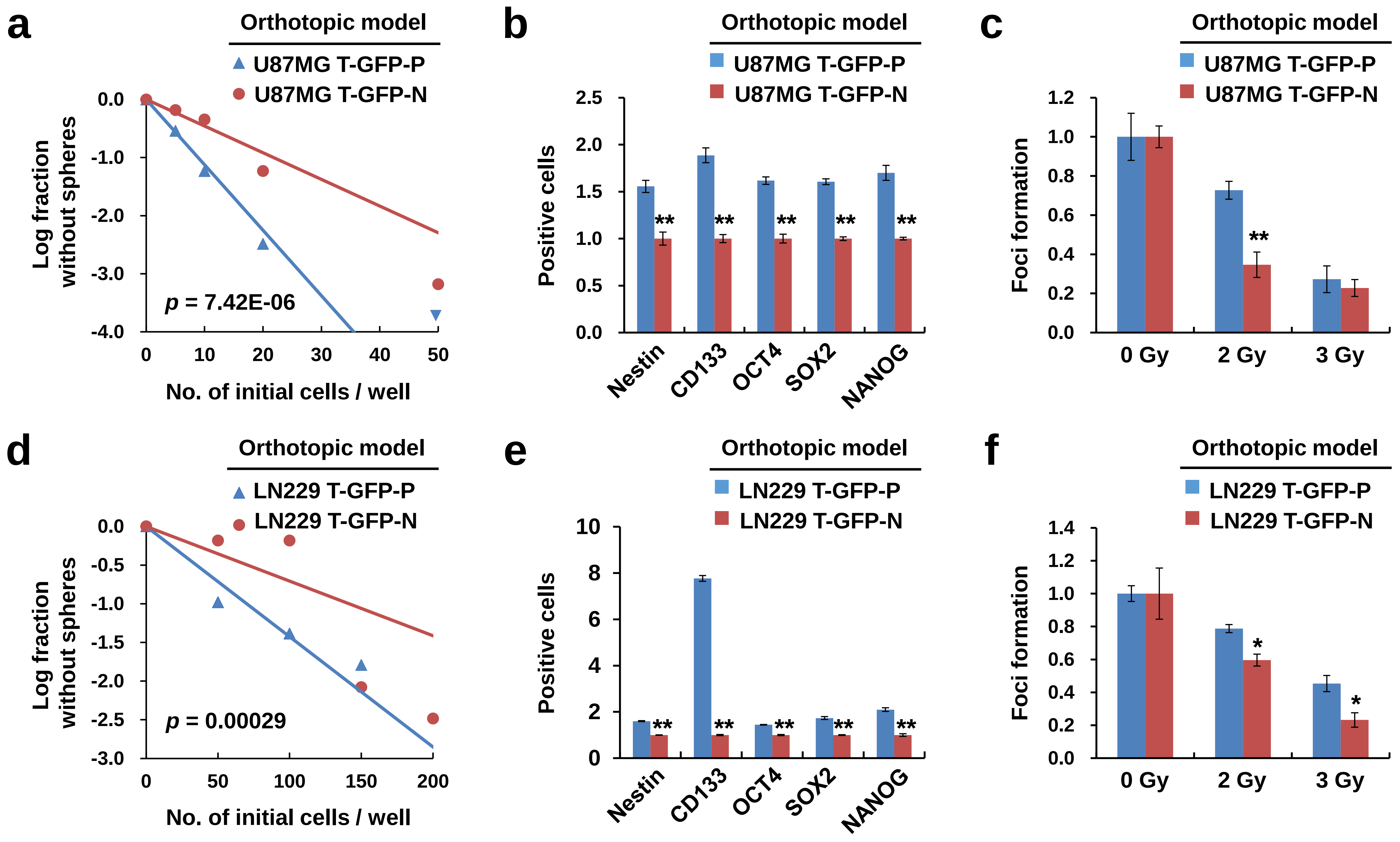

Supplementary Fig. 4 T-GFP-P cells derived from orthotopic co-injection tumors exhibit increased stem cell-like properties compared to T-GFP-N cells derived from the same tumors.

(a, d) *In vitro* limiting dilution assay showing stem cell sphere forming ability of T-GFP-P and T-GFP-N cells.

(b, e) FACS analysis showing the expression of several stem cell markers (Nestin, CD133, OCT4, SOX2, and NANOG) in T-GFP-P and T-GFP-N cells (**$p < 0.01$).

(c, f) Colony-formation assay showing the resistance of T-GFP-P and T-GFP-N cells to ionizing radiation (*$p < 0.05$, **$p < 0.01$).

Data in this figure are expressed as means ± SEM.

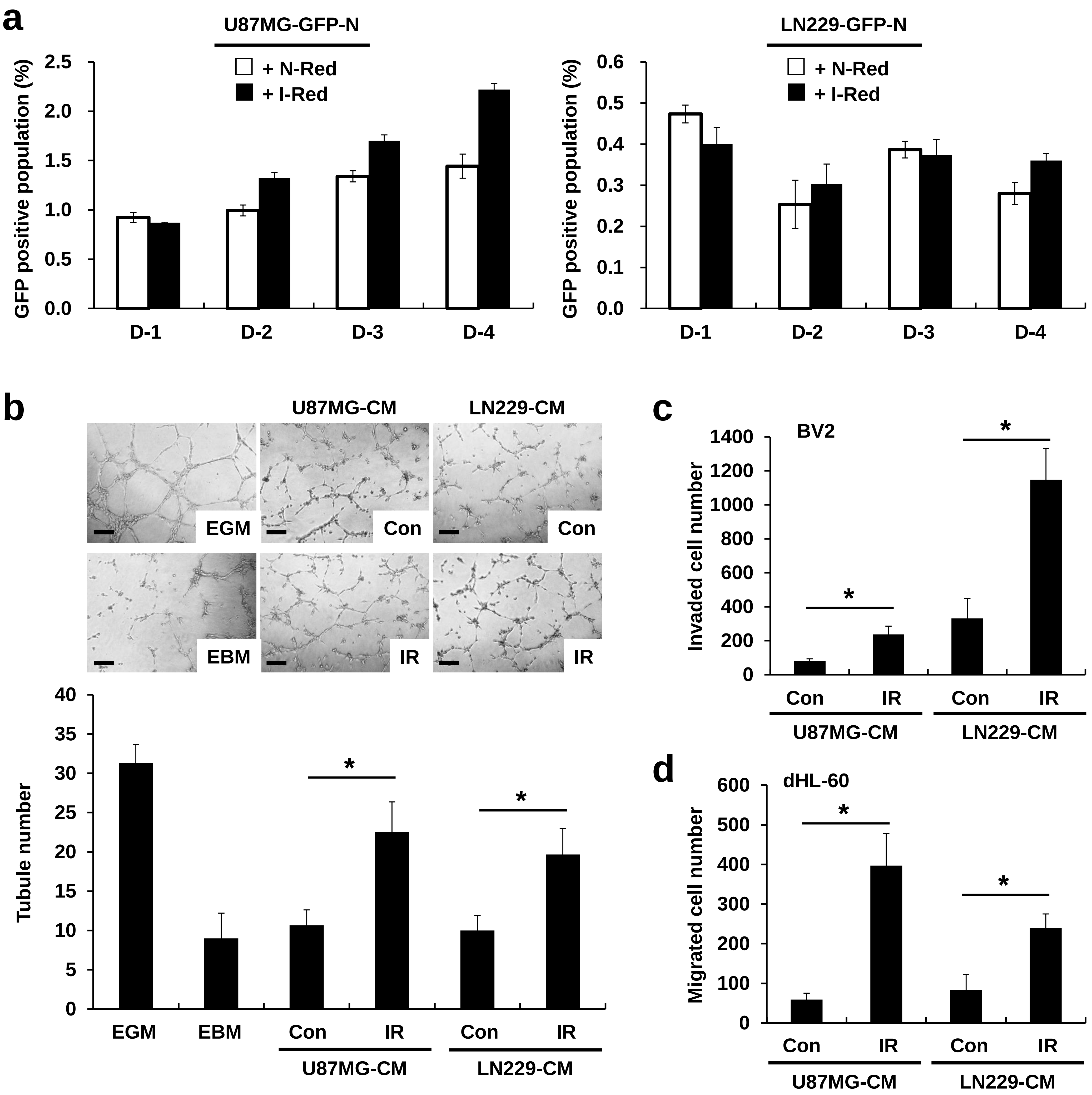

See the next page for figure legend

Supplementary Fig. 5 Irradiated glioma cells promote vessel formation and inflammatory cell recruitment *in vitro*.

(a) FACS analysis for detecting GFP-P cells derived from GFP-N cells that were co-cultured with N-Red cells or I-Red cells on the indicated day.

(b) *In vitro* tubule formation assay of HRECs incubated with CM harvested from N-Red cells or I-Red cells. Endothelial growth medium-2 (EGM-2) was used as a positive control and endothelial basal medium (EBM) was used as a negative control. Representative images showing tube formation (top) and quantification of the tube numbers (bottom, $*p < 0.05$). Scale bar represents 10 μm.

(c) Transwell invasion assay of BV2 microglial cells in the upper chamber with CM harvested from N-Red cells or I-Red cells in the bottom chamber. Quantification of the total number of invaded cells ($*p < 0.05$).

(d) Transwell migration assay of dHL-60 neutrophil cells in the upper chamber with CM harvested from N-Red cells and I-Red cells in the bottom chamber. Quantification of the total number of migrated cells ($*p < 0.05$).

Data in this figure are expressed as means ± SEM.

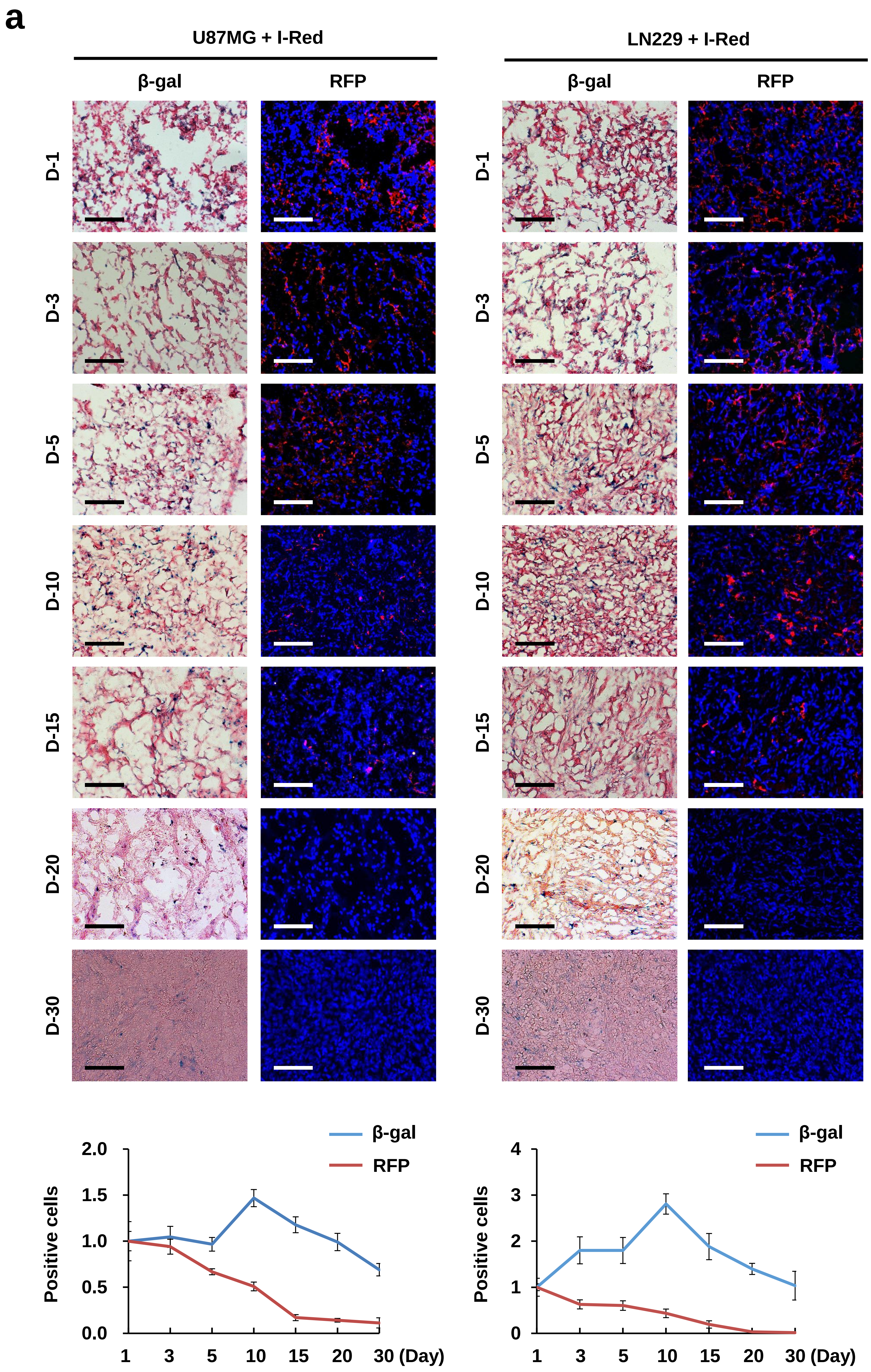
a
U87MG + I-Red
LN229 + I-Red
β-gal
RFP
D-1
D-3
D-5
D-10
D-15
D-20
D-30
Positive cells
2.0
1.5
1.0
0.5
0.0
4
3
2
1
0
1
3
5
10
15
20
30 (Day)

(Continued)

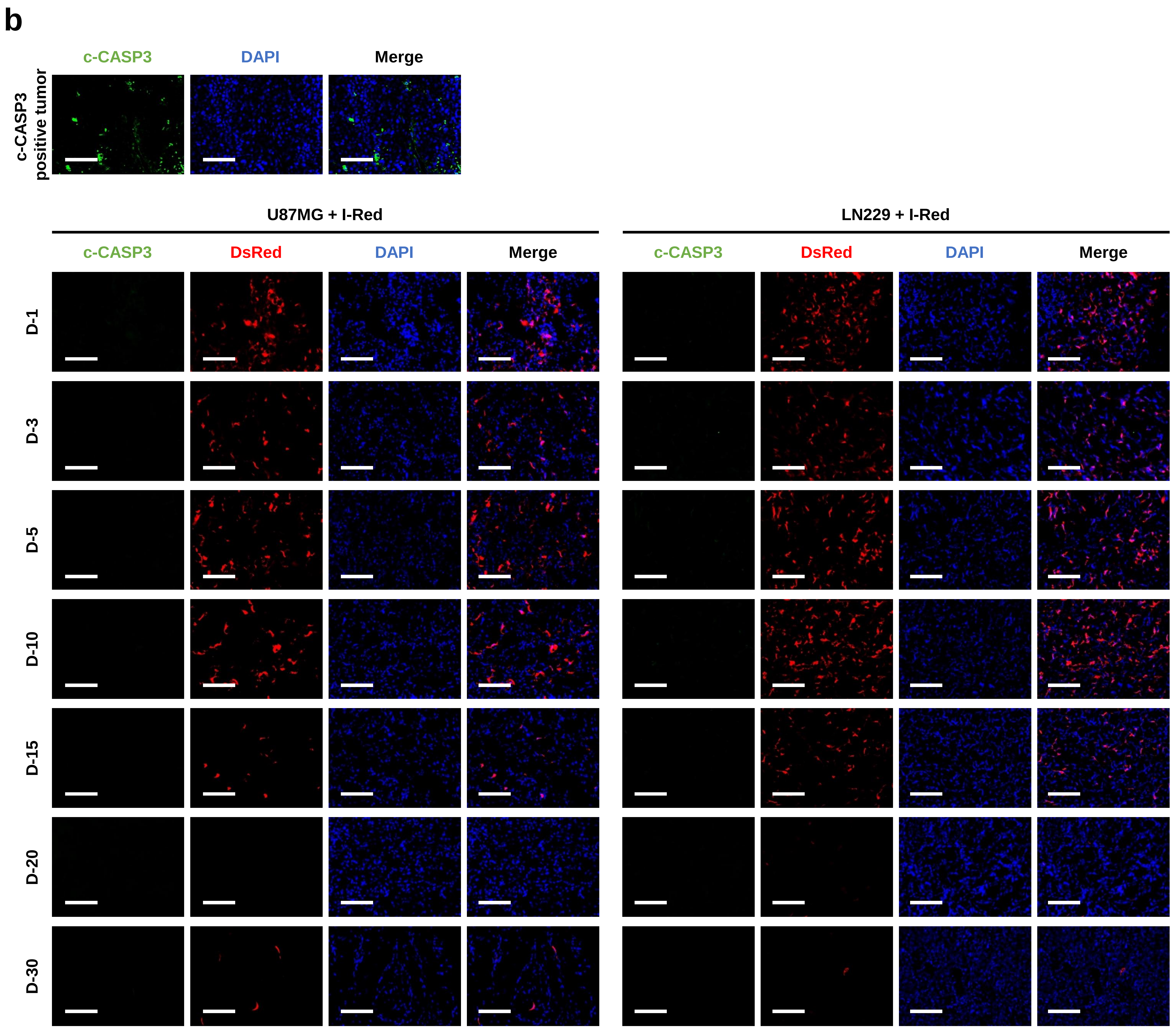

See the next page for figure legend

Supplementary Fig. 6 I-Red cells did not undergo cellular apoptosis, and instead achieved cellular senescence *in vivo*.

(a, b) SA-β-gal, anti-RFP and anti-c-CASP3 staining analysis of the tumor tissues harvested at the indicated day after subcutaneous injection of U87MG (1×10^6) + I-Red cells (2×10^6) or LN229 (1×10^6) + I-Red cells (2×10^6). (a) The majority of SA-β-gal$^+$ cells were co-stained with RFP, but unlike RFP$^+$ cells these cells had not completely disappeared by day 30. (b) c-CASP3$^+$ cells were not detected in the RFP$^+$ cells, but these cells were observed in stained GBM tissues treated with a cytotoxic drug (a positive control).

Data are expressed as means ± SEM. Scale bar represents 50 μm.

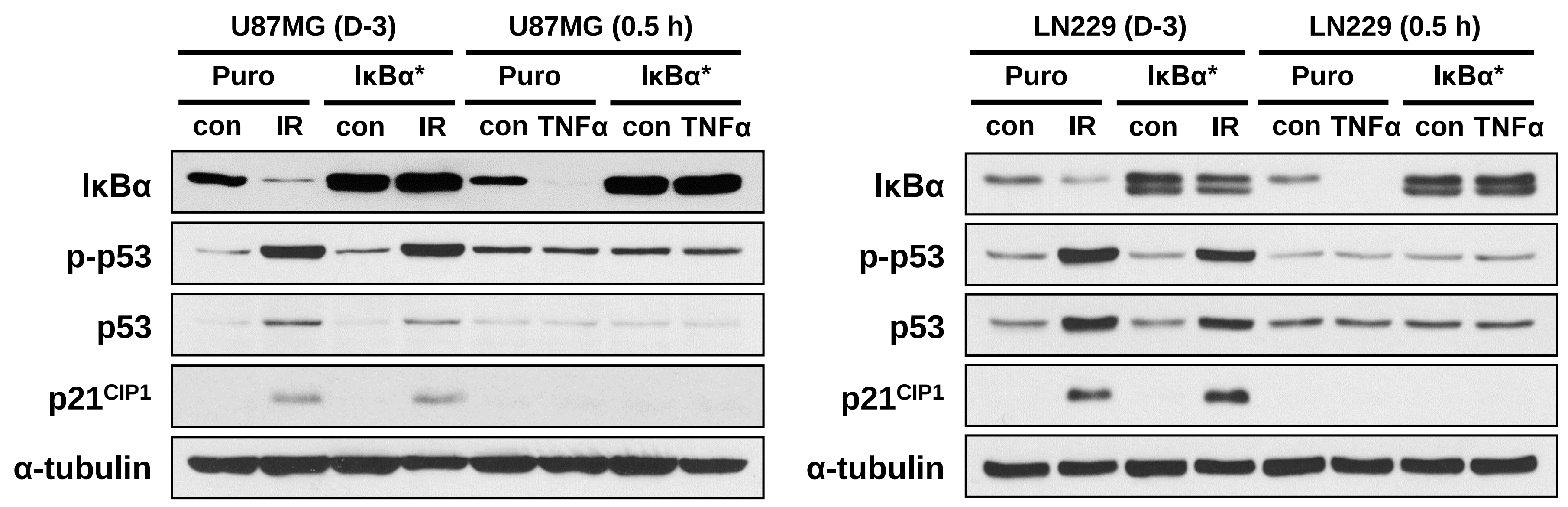

Supplementary Fig. 7 The super-repressor form of IκBα inhibits NFκB signaling, but not p53-p21^{Cip1} signaling, after irradiation.

Western blot analysis of U87MG and LN229 cells overexpressing mutant-IκBα (IκBα*) on day 3 following irradiation with 20 Gy. TNFα was used as a positive control for NFκB signaling.

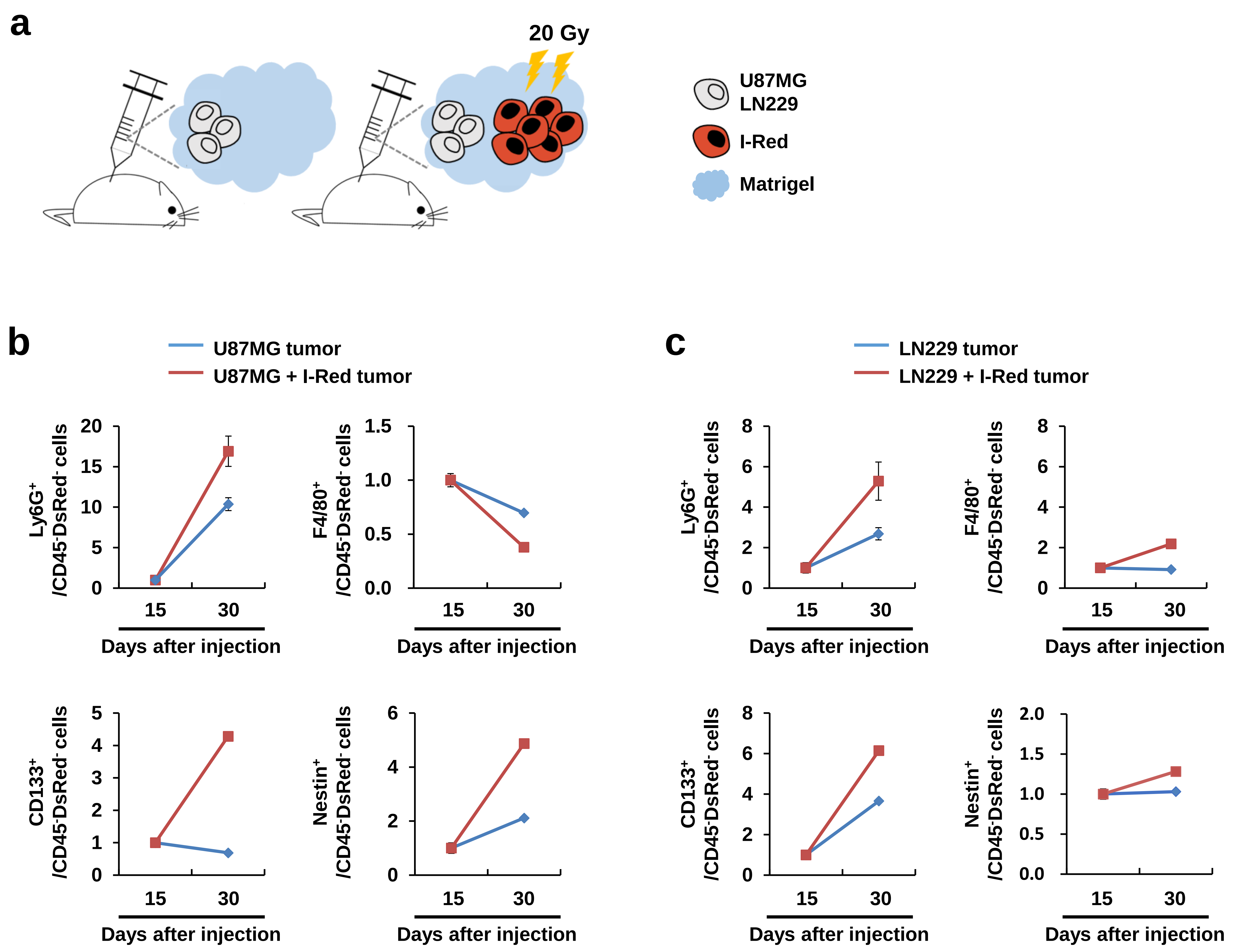

Supplementary Fig. 8 Increased recruitment of Ly6G+ inflammatory cells within xenograft tumors was associated with an increase in stem cell populations at early time points *in vivo*.

(a) Experimental scheme showing subcutaneous injection of non-irradiated glioblastoma cells (1 × 10^6) alone or a combination of non-irradiated glioblastoma cells (1 × 10^6) and I-Red (2 × 10^6) in the presence of Matrigel.

(b, c) FACS analysis indicating the cell populations positive for inflammatory cell markers ($CD45^+CD11b^+Ly6G^+$ or $CD45^+F4/80^+$) or stem cell markers ($CD45^-CD133^+$ or $CD45^-Nestin^+$) in the U87MG (b) and LN229 (c) xenograft tumors at days 15 and 30 post-injection.

Data in this figure are expressed as means ± SEM.

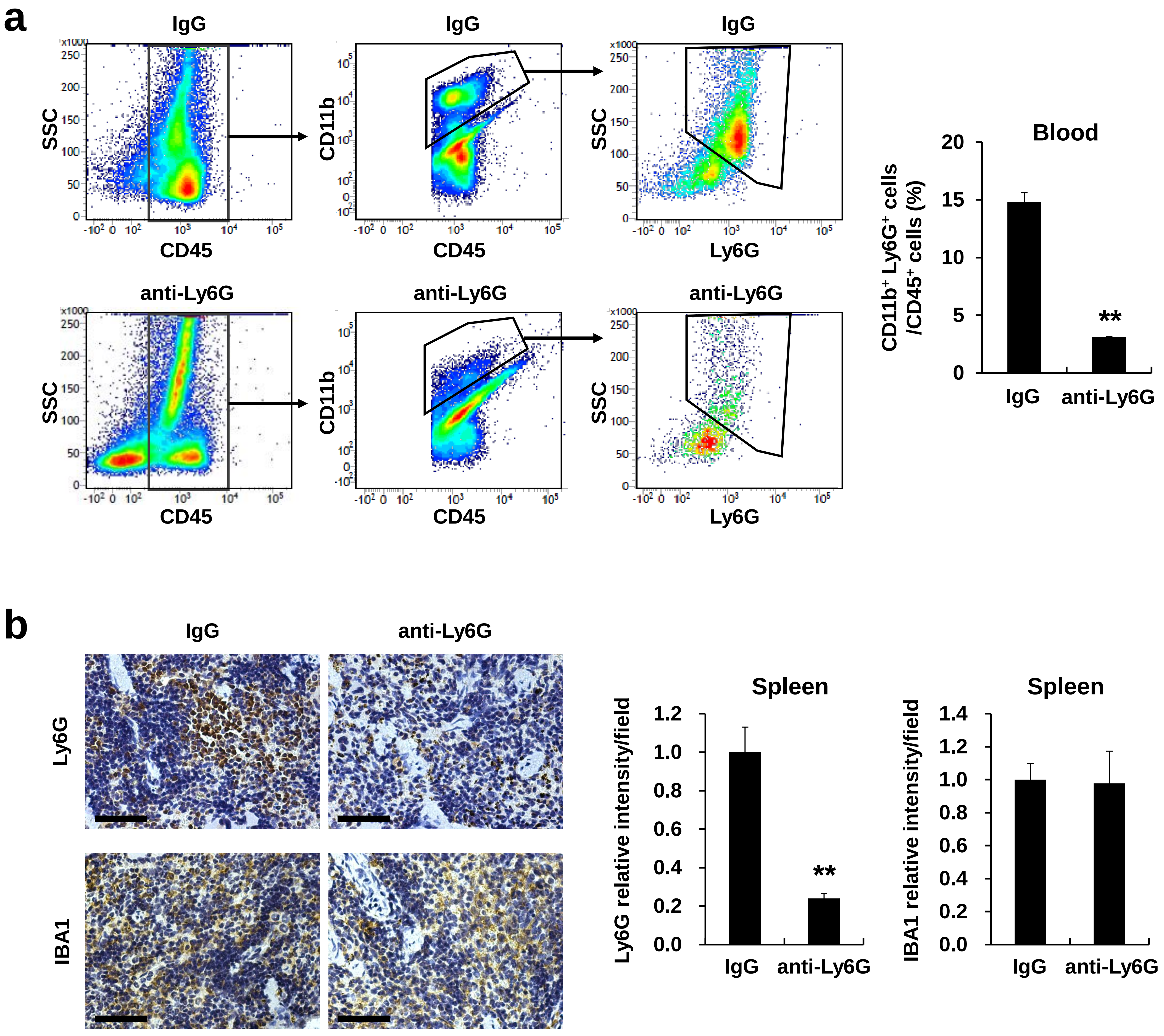

Supplementary Fig. 9 Treatment of anti-Ly6G antibody depleted neutrophils, but did not affect macrophages in the blood and spleen.

(a) Flow cytometry analysis of $CD45^{+}CD11b^{+}Ly6G^{+}$ neutrophils in the blood of the mice treated with either control IgG or anti-Ly6G (**p < 0.01; n = 3).

(b) Representative images and quantification of $Ly6G^{+}$ and $IBA1^{+}$ cells in the mouse spleen. (***p* < 0.01). Scale bar represents 50 μm.

Data in this figure are expressed as means ± SEM.

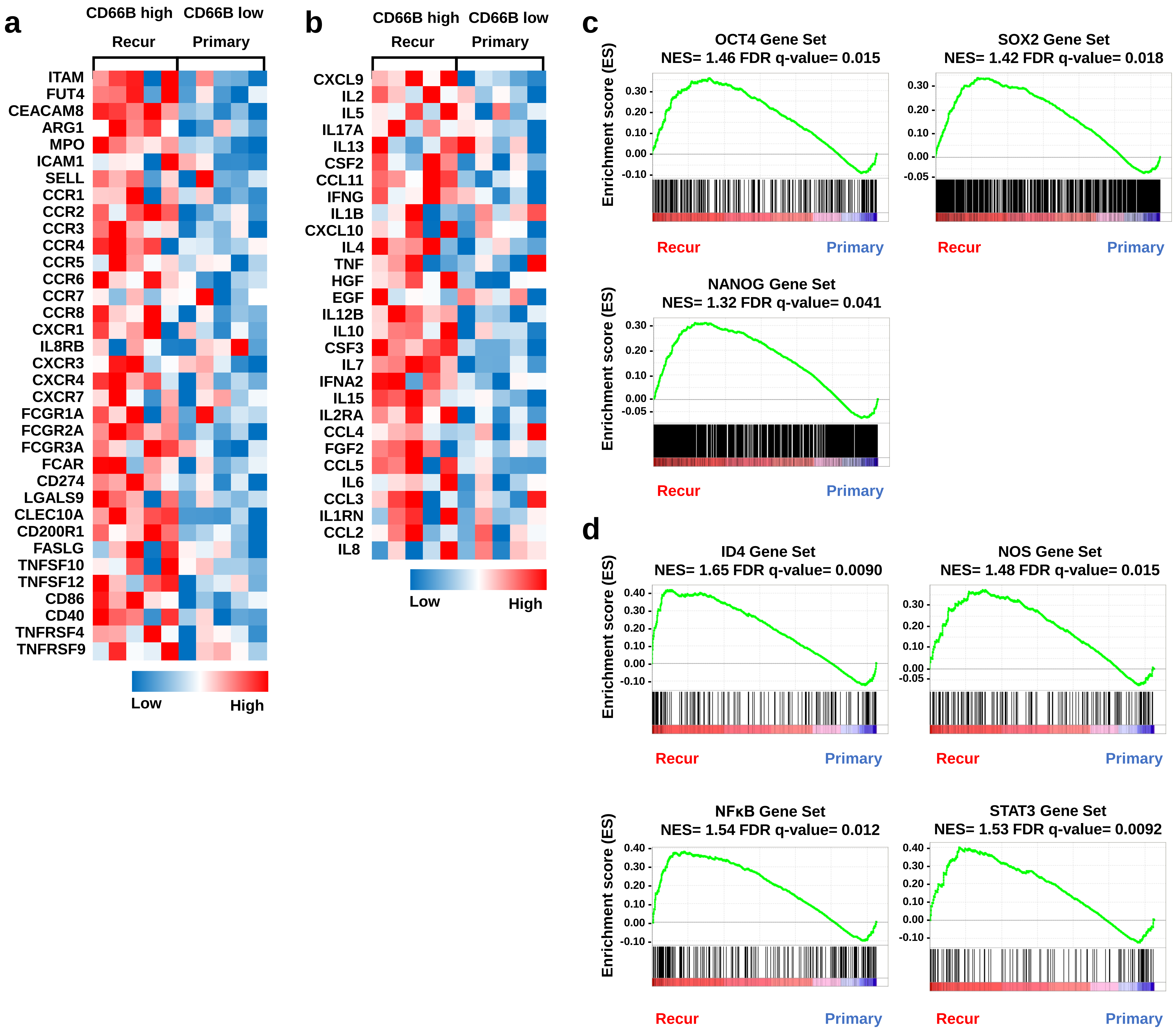

Supplementary Fig. 10 High CD66B expression correlates with TAN and dedifferentiation gene sets in recurrent GBM patients.

(a, b) Heatmap of TAN (A) and cytokine/chemokine (B) genes in recurrent GBM patient samples with high *CD66B vs.* those seen in primary GBM patient samples with low *CD66B*.

(c, d) GSEA data showing the enrichment of OCT4, SOX2 and NANOG gene sets (C) and ID4, NOS, NFκB, and STAT3 gene sets (D) in recurrent GBM patient samples with high *CD66B*.

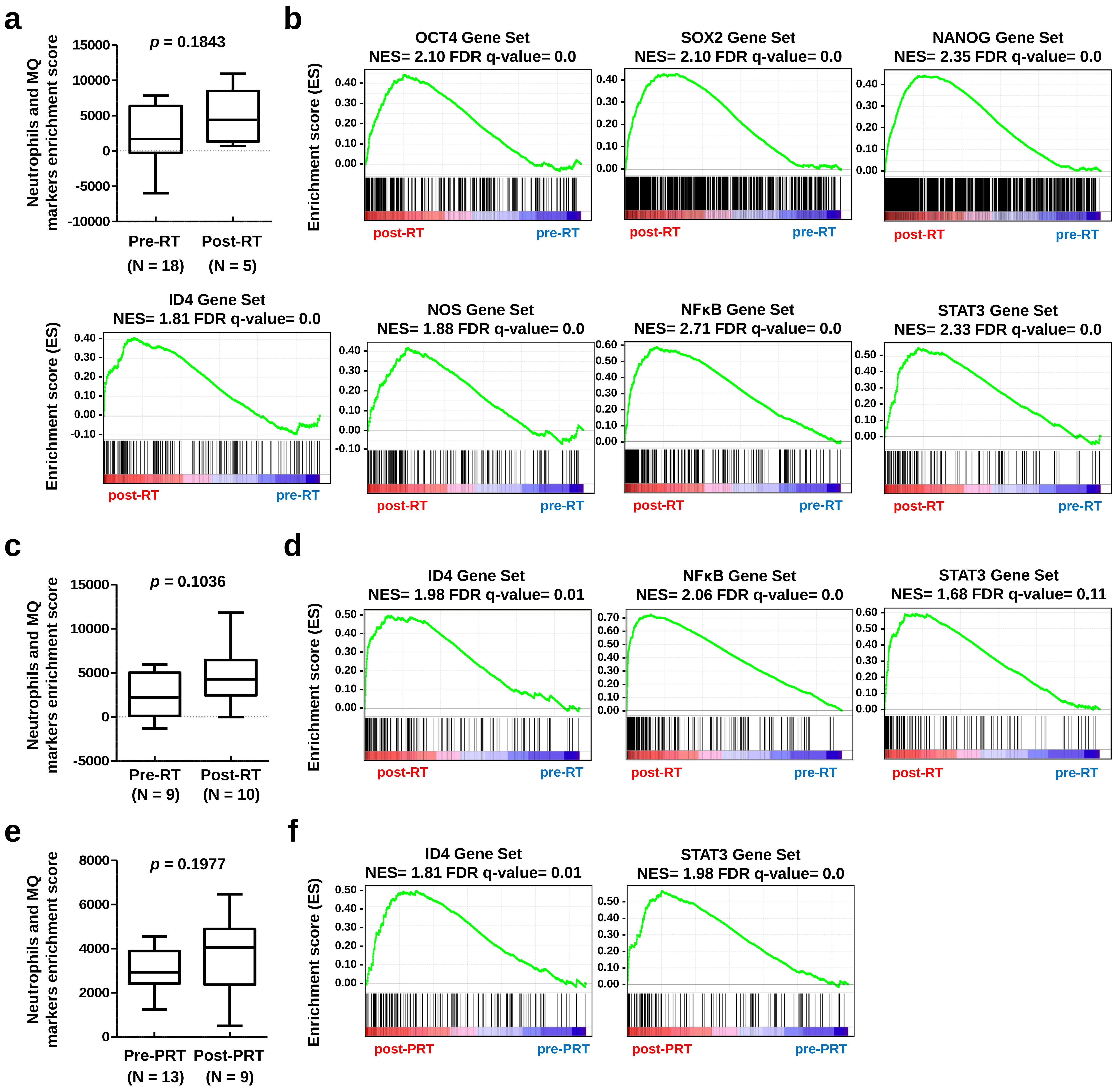
a
p = 0.1843
Neutrophils and MQ markers enrichment score
15000
10000
5000
0
-5000
-10000
Pre-RT (N = 18)
Post-RT (N = 5)
b
OCT4 Gene Set NES= 2.10 FDR q-value= 0.0
SOX2 Gene Set NES= 2.10 FDR q-value= 0.0
NANOG Gene Set NES= 2.35 FDR q-value= 0.0
ID4 Gene Set NES= 1.81 FDR q-value= 0.0
NOS Gene Set NES= 1.88 FDR q-value= 0.0
NFκB Gene Set NES= 2.71 FDR q-value= 0.0
STAT3 Gene Set NES= 2.33 FDR q-value= 0.0
Enrichment score (ES)
post-RT
pre-RT
c
p = 0.1036
Neutrophils and MQ markers enrichment score
15000
10000
5000
0
-5000
Pre-RT (N = 9)
Post-RT (N = 10)
d
ID4 Gene Set NES= 1.98 FDR q-value= 0.01
NFκB Gene Set NES= 2.06 FDR q-value= 0.0
STAT3 Gene Set NES= 1.68 FDR q-value= 0.11
Enrichment score (ES)
post-RT
pre-RT
e
p = 0.1977
Neutrophils and MQ markers enrichment score
8000
6000
4000
2000
0
Pre-PRT (N = 13)
Post-PRT (N = 9)
f
ID4 Gene Set NES= 1.81 FDR q-value= 0.01
STAT3 Gene Set NES= 1.98 FDR q-value= 0.0
Enrichment score (ES)
post-PRT
pre-PRT

Supplementary Fig. 11 Patients diagnosed with breast cancer or colorectal cancer exhibit an increased enrichment score of inflammatory cell markers and stem cell-like gene sets after radiotherapy.

(a, c, e) Box plots showing the enrichment score (ES) for a gene set of neutrophils and macrophage (MQ) markers between pre- and post-radiotherapy (RT) groups of breast cancer patients (a and c) and between pre- and post-preoperative radiotherapy (PRT) groups of colorectal cancer patients (e). The ES of neutrophils and MQ markers increased in patient samples taken post radiotherapy, but these increases did not have a significant *p* value.

(b, d, f) GSEA data representing the enrichment of OCT4, SOX2, NANOG, ID4, NOS, NFκB, and STAT3 gene sets in one group of post-RT breast cancer samples (b), ID4, NFκB, and STAT3 gene sets in another group of post-RT breast cancer samples (d), and ID4 and STAT3 gene sets in post-PRT colorectal cancer samples (f).

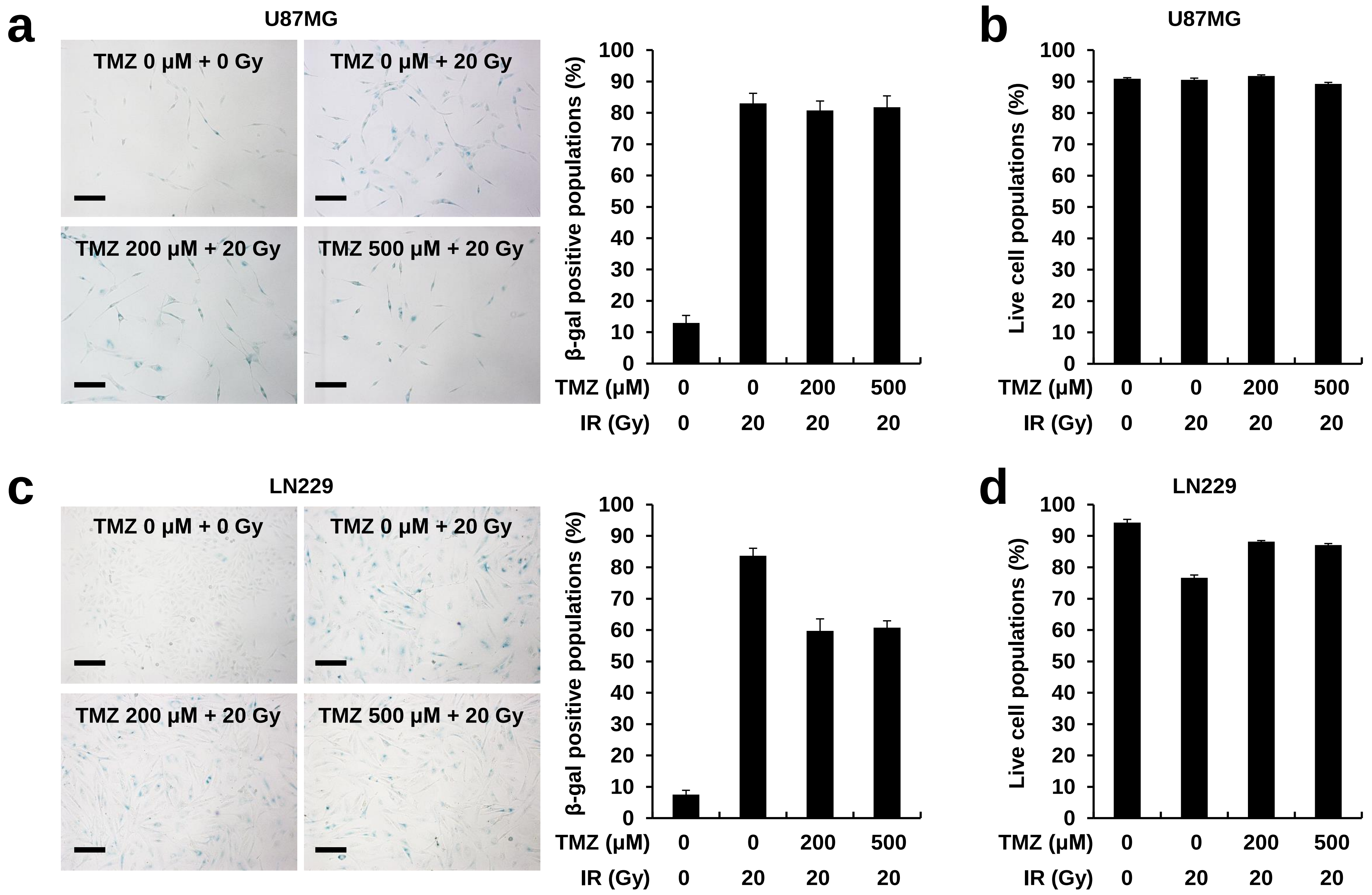

Supplementary Fig. 12 Combination treatment with TMZ and ionizing radiation induces senescence in glioma cells.

(a, c) The SA-β-gal staining assay of U87MG (A) and LN229 (C) cells treated with both TMZ (0, 200, and 500 μM) and irradiation at 20 Gy. TMZ did not alter cellular senescence after irradiation. Data are expressed as means ± SEM.

(b, d) Annexin V-PI staining assay of U87MG (B) and LN229 (D) cells treated with both TMZ (0, 200, and 500 μM) and irradiation at 20 Gy. Data are expressed as means ± SEM. Scale bar represents 50 μm.
